# Genetic mapping and candidate gene identification for key physiological traits associated with heat tolerance in wheat (*Triticum aestivum* L.) using a MAGIC population

**DOI:** 10.1371/journal.pone.0339966

**Published:** 2026-01-02

**Authors:** Ananta Bag, Hari Krishna, Vinodh Kumar P.N, Shiwani Meena, Narayana Bhat Devate, Sudhir Kumar, Ravindra Patil, Uday Govinda Reddy, Amit Kumar Singh, Badal Singh, Neelu Jain, Pradeep Kumar Singh, Gyanendra Pratap Singh

**Affiliations:** 1 Division of Genetics, Indian Agricultural Research Institute, New Delhi, India; 2 Division of Plant Physiology, Indian Agricultural Research Institute, New Delhi, India; 3 Genetics and Plant Breeding Group, Agharkar Research Institute, Pune, India; 4 Department of Genetics and Plant Breeding, University of Agricultural Sciences, Dharwad, India; 5 Division of Genomic Resources, ICAR-National Bureau of Plant Genetic Resources, New Delhi, India; 6 Division of Germplasm Evaluation, ICAR-National Bureau of Plant Genetic Resources, New Delhi, India; Institute of Genetics and Developmental Biology Chinese Academy of Sciences, CHINA

## Abstract

A comprehensive evaluation of 248 MAGIC population-derived wheat lines and their eight founder lines revealed significant genetic variation and adaptive diversity for key physiological traits- NDVI_1–3, SPAD chlorophyll content, canopy temperature (CT), and chlorophyll fluorescence (Fv/Fm UP and LW) under timely sown (TSIR) and late-sown (LSIR) irrigated conditions across multiple locations. Genotypes under TSIR exhibited higher canopy greenness, chlorophyll stability, and photosynthetic efficiency, whereas LSIR induced elevated CT and reduced SPAD, indicating genotypic differences in heat tolerance. Correlation and PCA analyses showed strong interrelationships among traits, with NDVI positively correlated with SPAD and Fv/Fm (r = 0.52-0.86) and negatively with CT (r = −0.13 to −0.60). PCA identified two principal adaptive axes- vigour/biomass (NDVI-CT) and pigment/stress (SPAD-Fv/Fm), explaining 65–82% of total variation. GWAS detected 54 significant marker-trait associations, predominantly on chromosome 5A, with major loci AX-95210025, AX-94980357, and AX-94448771 influencing NDVI, SPAD chlorophyll content, CT, and Fv/Fm (UP and LW). Favorable alleles enhanced canopy vigour and chlorophyll content while reducing CT, signifying integrated genetic control of photosynthetic resilience and thermal regulation. In-silico and gene regulatory network (GRN) analyses identified key heat-responsive candidate genes including *TraesCS5A02G078000* (Heat shock cognate 70 kDa protein), *TraesCS5A02G077900* (DnaJ co-chaperone), *TraesCS1A02G098800* (DUF4408 domain protein), *TraesCS7A02G143900* (Hydroxyproline O-arabinosyltransferase-like protein), and *TraesCS7B02G083500* (Small heat shock protein) that regulate chlorophyll maintenance, PSII repair, and canopy cooling under stress. Collectively, this study elucidates the genetic and physiological basis of heat tolerance in the MAGIC population and identifies robust targets for marker-assisted selection and genomic improvement of thermotolerant wheat.

## Introduction

Wheat (*Triticum aestivum* L.) is one of the world’s most important cereal crops, serving as a staple food for over half of the global population [1]. Consequently, achieving stable and enhanced wheat yields is essential to ensure global food security [2]. Although significant yield gains have been achieved since the Green Revolution, the current rate of increase remains insufficient to meet the rising food demand driven by rapid population growth and changing climatic conditions [3,4]. Among the various environmental challenges, high-temperature stress has emerged as a critical factor limiting wheat productivity worldwide [5].

To achieve sustainable yield improvement under such adverse conditions, identifying and selecting physiological traits associated with heat adaptation are of paramount importance. These traits provide opportunities to optimize genetic yield potential by improving processes such as assimilate partitioning, radiation use efficiency, and light interception [6]. Several physiological traits have been recognized as key contributors to heat tolerance in wheat, including higher leaf chlorophyll content measured as SPAD value, sustained leaf greenness assessed through the normalized difference vegetation index (NDVI), optimum canopy temperature (CT), and efficient photosynthetic performance indicated by Fv/Fm. Despite their importance, limited knowledge of the genetic basis of these traits constrains their effective use in breeding programs [7,8]. Identifying novel genetic loci or quantitative trait loci (QTL) associated with these physiological traits under heat stress and integrating them into marker-assisted selection (MAS) can lead to cumulative genetic gains in yield. This approach forms the foundation of marker-assisted physiological trait breeding, offering a promising pathway for developing heat-tolerant wheat cultivars [9].

Genome-wide association studies (GWAS) have emerged as a powerful approach for dissecting the genetic basis of complex physiological traits associated with stress tolerance in wheat. Unlike traditional bi-parental QTL mapping, which is often limited by low resolution and population specificity, GWAS leverages the natural allelic diversity present in diverse germplasm panels, offering higher mapping precision and broader applicability [10]. Several GWAS investigations have identified significant marker-trait associations (MTA) for key physiological traits such as normalized difference vegetation index (NDVI), canopy temperature (CT), SPAD chlorophyll content, and chlorophyll fluorescence parameters (Fv/Fm) under heat stress conditions. NDVI-associated MTA were detected on chromosomes 1A, 4B, 4D, 7B, and 7D [11], 1A and 4B [12], and 4B and 4D [13], highlighting genomic regions linked to the stay-green phenotype [14]. Multiple MTA for canopy temperature (CT) have been reported on chromosomes 2B, 5A, 7B, 2D, 1D, 4A, and 5D, and for SPAD chlorophyll content on 3D, 4A, 7A, and 7D, indicating their significant role in maintaining photosynthetic efficiency and chlorophyll stability under thermal stress [15]. In addition, chlorophyll fluorescence parameters, such as the maximum quantum yield of PS-II (Fv/Fm), provide valuable information on the effects of high temperature on photochemical efficiency and the underlying tolerance mechanisms. MTA for Fv/Fm have been identified on chromosomes 3B, 6D, 5A, and 7B, suggesting the involvement of multiple loci in PS-II thermotolerance [16]. The development of Multi-parent Advanced Generation Inter-Cross (MAGIC) populations has emerged as a significant advancement in plant genetics and breeding, providing next-generation mapping resources that overcome the limitations of conventional biparental and association mapping populations [17–20]. Originally demonstrated in the mouse collaborative cross [21], the MAGIC approach was subsequently adapted for crop species to facilitate precise QTL mapping and enhance varietal development.

The present study aimed to dissect the genetic basis of key physiological traits, including NDVI, SPAD chlorophyll content, canopy temperature (CT), and chlorophyll fluorescence (Fv/Fm), under heat stress conditions in wheat. A genome-wide association study (GWAS) was performed using the *wheat 35K Affymetrix SNP array* genotyping data in a MAGIC population, which captured a broad spectrum of genetic diversity and enabled high-resolution mapping of loci controlling these physiological traits. In addition, *In-silico* candidate gene identification and expression analyses were conducted to pinpoint potential genes associated with these traits under heat stress conditions.

## Materials and methods

### Plant materials and growth conditions

In this study, 248 MAGIC population-derived lines, along with their eight founder lines, were evaluated for physiological traits. The founder lines represented diverse wheat-growing regions and adaptation conditions across India: HD 3086 and HD 3043 from the North Western Plains Zone (NWPZ) performed under timely sown irrigated and restricted irrigated conditions, respectively; HD 2985 and HI 1563 from the North Eastern Plains Zone (NEPZ) excelled under late sown irrigated conditions; HD 2932 and HI 1544 from the Central Zone were adapted to late sown and timely sown irrigated conditions, respectively; VL 907 from the Northern Hill Zone suited late sown irrigated and rainfed conditions, while GW 322 from the Peninsular Zone thrived under timely sown irrigated conditions.

The MAGIC population was developed beginning in Rabi 2013−14, and the 248 MAGIC population-derived F₉ RILs and their founder lines were evaluated across three location- IARI, Delhi; UAS, Dharwad; and ARI, Pune during 2024−25. Field experiments were conducted on institutional research farms, and official permission for field site access was granted by the Division of Genetics, ICAR–Indian Agricultural Research Institute (IARI), New Delhi; the Department of Genetics and Plant Breeding, University of Agricultural Sciences (UAS), Dharwad; and the Genetics and Plant Breeding Group, Agharkar Research Institute (ARI), Pune. Field trials were conducted under timely sown irrigated (TSIR) and late sown irrigated (LSIR) conditions to assess physiological traits. To minimize field variability, trials were conducted using an alpha-lattice design comprising 16 blocks of 16 plots each.

Physiological traits were measured systematically under TSIR and LSIR with clearly defined intervals to enable comparative assessment of crop growth and development across the two environments. NDVI (Normalized Difference Vegetation Index) was recorded at three stages NDVI_1 during vegetative growth (stem elongation/pre-booting), NDVI_2 at heading/anthesis, and NDVI_3 during grain filling to early maturity using a handheld *GreenSeeker®* (Trimble Inc., USA) held 0.6-1.0 m above the canopy, with measurements taken at 15–20 days interval across both sowing conditions and values recorded on a 0–1 scale. Chlorophyll content (SPAD) of the flag leaf was measured using a SPAD-502 Plus meter (Konica Minolta, Japan) between heading and grain filling at 10–15 days intervals, with two readings per condition at each location and values recorded on a 0–100 scale. Canopy temperature (CT) was measured during grain filling using an infrared *Thermal imager* (Huazhong, China) positioned 0.5-1.0 m above the canopy, recorded at 10 days interval and expressed in°C. Chlorophyll fluorescence (Fv/Fm) for upper (Fv/Fm_UP) and lower (Fv/Fm_LW) leaves was assessed using a *Floupen FP 110* (Photon Systems Instruments, Czech Republic) from booting to heading after dark-adapting leaves for 10–20 minutes, measured at 7–10 days intervals and expressed as a ratio (0–1) reflecting photosystem II efficiency. These defined measurement intervals and detailed methodologies ensure robust comparison of physiological responses between TSIR and LSIR conditions.

Meteorological records from all three experimental sites showed clear temperature differences between TSIR and LSIR conditions at the time of physiological trait measurements. At Delhi, maximum temperatures during NDVI, CT, SPAD, and Fv/Fm assessments ranged from 22.2 to 34.6°C under TSIR, while LSIR recordings occurred under higher maximum temperatures ranging from 25.0 to 36.0°C, confirming the intended heat-stress contrast. Similarly, at Dharwad, maximum temperatures increased from 29.2 to 33.2°C in TSIR and from 28.8 to 34.0°C in LSIR across NDVI, CT, and SPAD measurements. At Pune, maximum temperatures ranged from 30.7 to 34.7°C in TSIR and from 32.8 to 36.0°C in LSIR. These higher maximum temperatures in LSIR across all sites validate the heat-stress conditions and support comparative evaluation of trait responses under contrasting thermal environments. Meteorological data (maximum, minimum, and mean temperatures) corresponding to each physiological trait measurement for all three sites are provided in S4-S6 Tables, and the full daily meteorological records for each location are provided in S7-S9 Tables.

### SNP genotyping

High-quality genomic DNA was isolated from young leaf tissues of 248 MAGIC population lines and their eight founder lines using the CTAB method. DNA quality and integrity were verified by agarose gel electrophoresis, and concentrations were measured with a NanoDrop spectrophotometer. Only samples with concentrations above 25 ng/μl and minimal degradation were selected for genotyping to ensure reliable downstream analyses. Genotyping was performed using the *35K Axiom® Wheat Breeder’s Array* (*Affymetrix*), providing high-density genome-wide coverage encompassing both functional and neutral markers suitable for association mapping. Hybridization, washing, staining, and scanning were conducted on the *Affymetrix GeneTitan*® system following the manufacturer’s standard protocol for DNA amplification, fragmentation, and hybridization. Initial allele calling and quality control were carried out using Axiom Analysis Suite software, following the *Axiom® Best Practices Genotyping Workflow* (https://documents.thermofisher.com/TFS-Assets/LSG/manuals/MAN0018363-AxiomDataAnalysis-UG-RUO.pdf.). After stringent filtering for missing data (<10%) and minor allele frequency (>5%), a total of 11,574 high-quality SNPs were retained. These markers were evenly distributed across all 21 wheat chromosomes, ensuring robust genome coverage for genome-wide association studies (GWAS), allelic effect analysis, and in silico functional annotation.

### Statistical analysis

Phenotypic data for physiological traits were systematically analyzed using R software. For each trait under timely sown irrigated (TSIR) and late sown irrigated (LSIR) conditions across the three locations (Delhi, Dharwad, and Pune), key descriptive parameters including mean, standard error (SE), standard deviation (SD), minimum, maximum, least significant difference (LSD), coefficient of variation (CV), mean sum of squares (MSS), significance, and broad-sense heritability (h^2^) were computed using the *agricolae* package in R. Best Linear Unbiased Predictors (BLUPs) and adjusted means were estimated with Meta-R software [22] by fitting models that accounted for replication, block, genotype, and genotype-by-environment (G × E) interactions. Furthermore, pairwise Pearson correlation coefficients (r) were calculated to evaluate linear relationships among traits, and correlation matrices were visualized using the *corrplot* package in R. Principal component analysis (PCA) was also performed using *FactoMineR* and *factoextra* in R to investigate patterns of trait variation and to identify the major traits contributing to variability under both stress and non-stress conditions.

### Genome wide association study (GWAS)

Genome-wide association analysis (GWAS) was performed in R using GAPIT version 3, employing the BLINK model, Bayesian-information and Linkage-disequilibrium Iteratively Nested Keyway [23], to detect quantitative trait nucleotides (QTNs) while accounting for population structure and linkage disequilibrium to minimize false positives. The analysis utilized high-quality filtered SNP markers together with BLUP values calculated across locations and sowing conditions as input, following the GAPIT user guidelines (https://zzlab.net/GAPIT/gapit_help_document.pdf). Model adequacy was assessed using quantile–quantile (Q-Q) plots, where conformity along the diagonal indicated proper model fit, and deviations at the tail suggested significant associations. Significant marker–trait associations were identified at a threshold of *-log₁₀(p) > 3 (p < 0.001)*, and the results were visualized with Manhattan plots to illustrate the genomic positions and significance levels of the associated SNPs.

### Allelic effect analysis of significant SNPs

Allelic effect analysis was conducted to evaluate the functional contribution of significant SNPs associated with NDVI, canopy temperature (CT), SPAD chlorophyll content, and Fv/Fm in the MAGIC population. For each SNP identified through GWAS, the population was grouped according to allelic classes, and the mean trait values of each class were compared to estimate the phenotypic effects of favourable versus unfavourable alleles. Standard deviations were calculated, and *t*-tests were performed to assess the statistical significance of differences between allelic classes. To ensure accurate and robust estimation of allelic effects, BLUP-adjusted trait values across all environments were used under both timely sown irrigated (TSIR) and late sown irrigated (LSIR) conditions.

### *In-silico* candidate gene analysis

Potential candidate genes associated with trait-linked SNPs were identified by performing BLAST searches using the sequences of significant markers on the *Ensembl Plants platform* (https://plants.ensembl.org/Triticum_aestivum/Tools/Blast) against the bread wheat reference genome *IWGSC RefSeq v1.0* [24], allowing precise genomic localization of SNPs. To capture genes within the typical linkage disequilibrium (LD) range in wheat, genomic regions spanning ±100 kb around each associated SNP were examined using the “*Region Comparison*” tool in *Ensembl Plants*, and putative candidate genes along with their transcript IDs were retrieved. Among the genes located in this region, we prioritized those with annotated functions related to heat stress response, photosynthesis, chlorophyll metabolism, membrane stability, antioxidant activity, or plant developmental processes. Functional annotation of the identified genes was performed using *UniProt*, providing insights into the encoded proteins and their biological roles.

### *In-silico* candidate gene expression analysis

The expression profiles of candidate genes associated with physiological traits, including NDVI, canopy temperature (CT), SPAD chlorophyll content, and Fv/Fm, were analyzed in the wheat MAGIC population using publicly available transcriptome datasets. The analysis was conducted under two contrasting conditions: timely sown irrigated (TSIR) and late sown irrigated (LSIR), representing normal and heat stress environments, respectively. Gene expression levels, expressed as transcripts per million (TPM), were obtained from the *Wheat Expression Browser* [25].

### Gene regulatory network construction and analysis

The functional and regulatory roles of candidate genes associated with physiological traits in the MAGIC population were investigated using gene regulatory network (GRN) analysis via the *KnetMiner Plants Lite platform* (https://app.knetminer.com/plants-lite/Triticum_aestivum). This platform enabled the identification, extraction, and visualization of gene-level connections, including co-expression relationships, protein–protein interactions, and pathway associations involved in hormone signaling and stress responses. The analysis integrated multi-omics datasets from various public sources. Additionally, *KnetMiner*’s ability to incorporate comparative information from model species such as *Arabidopsis thaliana*, *Oryza sativa*, *Brassica napus* and *Camelina sativa* was utilized to reveal evolutionarily conserved regulatory networks that influence physiological processes.

## Results

### Phenotypic evaluation

A comprehensive analysis of phenotypic variation among the 248 MAGIC population-derived lines and their eight founder lines revealed substantial diversity across environments and locations. Under timely sown irrigated (TSIR) and late sown irrigated (LSIR) conditions, significant differences (p < 0.001) were observed for all physiological traits- NDVI, SPAD chlorophyll content, canopy temperature (CT), and Fv/Fm (UP and LW), indicating substantial genetic variability among genotypes (Table 1).

Under Timely Sown Irrigated (TSIR) conditions, NDVI values (NDVI_1, NDVI_2 and NDVI_3) ranged from 0.24 to 0.84 across stages while SPAD (chlorophyll content) readings ranged from 29.9 to 48.0 indicated moderate to high chlorophyll content. Canopy temperature (24.2–27.8°C) reflected favourable conditions with minimal heat stress, and Fv/Fm values ranged from 0.45 to 0.77 demonstrated good photosystem II efficiency. Under Late Sown Irrigated (LSIR) conditions, NDVI ranged from 0.13 to 0.84, showing reduced canopy vigour and accelerated senescence due to higher temperature stress, while SPAD (chlorophyll content) values ranged from 26.3 to 53.6. Canopy temperature (CT) was notably higher (25.3–36.5°C), confirming heat stress, and Fv/Fm values (0.39–0.74) suggested reduced photosynthetic efficiency. Overall, TSIR conditions favoured higher NDVI and Fv/Fm with lower CT as compared to LSIR conditions.

**Table 1:** Descriptive statistics of physiological traits of MAGIC population-derived lines evaluated under timely sown irrigated (TSIR) and late sown irrigated (LSIR) conditions across three locations.

Traits	Env.	Mean	SE	SD	Min	Max	h^2^	LSD	CV	MSS	SIGN^a^
CT	LS_DL	31.050	0.135	2.167	25.490	36.523	0.780	1.083	2.640	9.487	***
CT	TS_DHAR	24.212	0.051	1.148	21	27.300	0.710	1.155	2.791	1.922	***
CT	LS_DHAR	25.324	0.049	1.103	22.500	28	0.689	1.038	2.440	1.439	***
CT	TS_PUNE	27.762	0.052	1.179	25.530	30.733	0.739	1.226	2.545	2.030	***
CT	LS_PUNE	29.433	0.042	0.671	27.060	30.680	0.733	1.176	2.296	1.890	***
Fv/Fm LW	TS_DL	0.632	0.002	0.052	0.447	0.740	0.661	0.046	3.848	0.005	***
Fv/Fm LW	LS_DL	0.591	0.004	0.057	0.393	0.740	0.751	0.055	4.774	0.008	***
Fv/Fm UP	TS_DL	0.701	0.002	0.037	0.600	0.767	0.617	0.041	3.810	0.002	***
Fv/Fm UP	LS_DL	0.680	0.001	0.019	0.622	0.723	0.639	0.041	3.716	0.002	***
NDVI 1	TS_DL	0.756	0.002	0.041	0.620	0.830	0.712	0.039	3.029	0.003	***
NDVI 1	LS_DL	0.706	0.001	0.024	0.632	0.773	0.583	0.057	5.308	0.004	***
NDVI 1	TS_DHAR	0.712	0.002	0.052	0.600	0.810	0.842	0.049	3.702	0.005	***
NDVI 1	LS_DHAR	0.741	0.002	0.042	0.640	0.830	0.704	0.047	3.803	0.003	***
NDVI 1	TS_PUNE	0.858	0.002	0.041	0.745	0.830	0.704	0.045	3.063	0.002	***
NDVI 1	LS_PUNE	0.806	7 E-04	0.012	0.761	0.837	0.625	0.026	2.028	7E-04	***
NDVI 2	TS_DL	0.512	0.004	0.095	0.240	0.710	0.699	0.105	12	0.013	***
NDVI 2	LS_DL	0.531	0.003	0.055	0.379	0.667	0.811	0.076	7.913	0.009	***
NDVI 2	TS_DHAR	0.617	0.003	0.068	0.440	0.790	0.777	0.069	6.280	0.007	***
NDVI 2	LS_DHAR	0.669	0.003	0.059	0.540	0.800	0.852	0.055	4.456	0.006	***
NDVI 2	TS_PUNE	0.735	0.004	0.090	0.490	0.690	0.667	0.100	8.278	0.012	***
NDVI 2	LS_PUNE	0.665	0.002	0.040	0.543	0.749	0.572	0.099	9.778	0.011	***
NDVI 3	TS_DL	0.302	0.004	0.096	0.090	0.570	0.614	0.103	21	0.013	***
NDVI 3	LS_DL	0.342	0.006	0.093	0.127	0.573	0.833	0.120	19	0.026	***
NDVI 3	TS_DHAR	0.342	0.005	0.123	0.101	0.613	0.836	0.118	18.770	0.026	***
NDVI 3	LS_DHAR	0.294	0.005	0.115	0.120	0.610	0.838	0.110	20.470	0.023	***
NDVI 3	TS_PUNE	0.534	0.007	0.152	0.240	0.550	0.884	0.130	12.760	0.040	***
NDVI 3	LS_PUNE	0.512	0.005	0.080	0.347	0.610	0.649	0.169	20.380	0.032	***
SPAD	TS_DL	29.898	0.585	13.240	3.100	57.933	0.846	12.310	22	294	***
SPAD	LS_DL	42.885	0.427	6.826	26.300	53.550	0.859	7.785	9.845	128	***
SPAD	TS_DHAR	45.616	0.153	3.455	36.800	55.450	0.766	3.618	5	18.84	***
SPAD	LS_DHAR	47.990	0.129	2.916	39.750	55.550	0.750	3.152	3.784	13.410	***
SPAD	TS_PUNE	47.958	0.097	2.196	41.700	53.200	0.664	2.461	3.138	7.040	***
SPAD	LS_PUNE	48.638	0.108	1.730	44	53.194	0.728	2.997	3.622	11.690	***

TS, timely sown irrigated (TSIR) condition; LS, late sown irrigated (LSIR) condition; SE, Standard error; SD, standard deviation; LSD, least significant difference; CV, coefficient of variation; MSS, mean sum of squares; h^2^, broad-sense heritability.

^a^Significance levels: ***p < 0.001, **p < 0.01, *p < 0.05.

### Correlation among physiological traits

**Fig 1:**
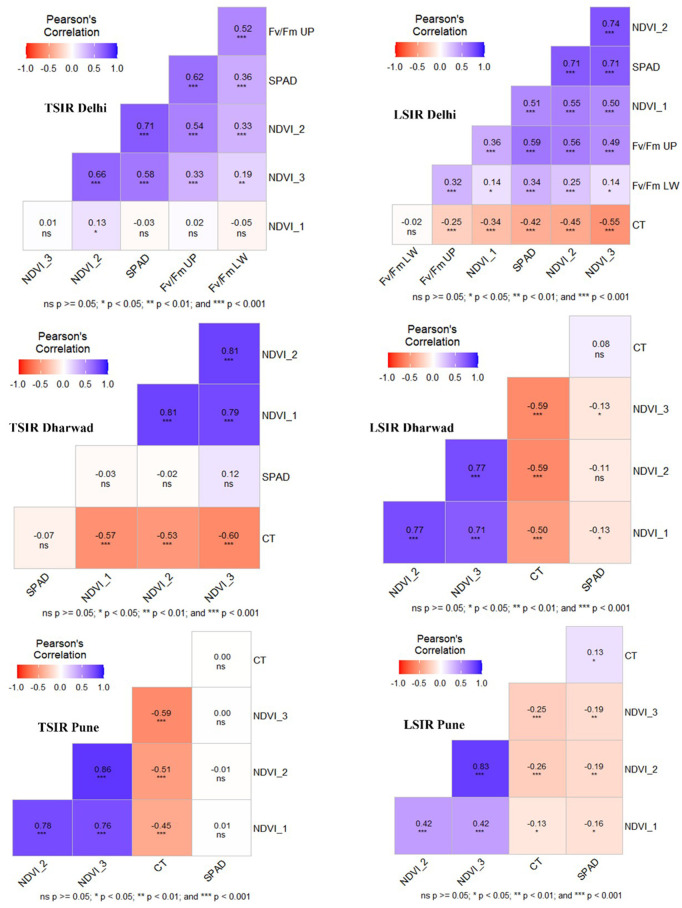
Correlation among physiological traits evaluated across three locations: IARI-Delhi; UAS-Dharwad; and ARI-Pune under TSIR and LSIR conditions. Positive and negative correlations are represented by blue and orange colour gradients, respectively.

NDVI (Normalized Difference Vegetation Index) traits consistently exhibited strong inter correlations across all study locations. A critical finding was the clear positive link between Fv/Fm (photosynthetic efficiency) and canopy greenness, evidenced by its uniform positive correlation with SPAD chlorophyll content and NDVI_2 (r = 0.52-0.62). Canopy temperature reliably served as a key stress indicator, showing a negative correlation (r = −0.13 to −0.60) with both NDVI and Fv/Fm, implying that cooler canopies relate to higher greenness and photosynthetic efficiency. However, the SPAD correlation with NDVI demonstrated significant environmental influences, being significant at Delhi (0.62-0.71) but non-significant at Dharwad and Pune (Fig 1).

### Principle component analysis (PCA)

**Fig 2:**
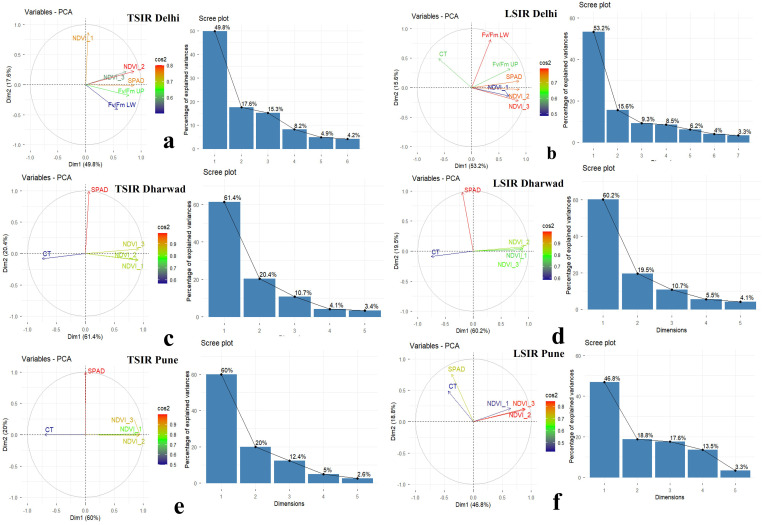
Principal component analysis (PCA) plots for physiological traits of MAGIC lines under different environments. **(a)** Delhi-TSIR, **(b)** Delhi-LSIR, **(c)** Dharwad-TSIR, **(d)** Dharwad-LSIR, **(e)** Pune-TSIR, and **(f)** Pune-LSIR.

The multivariate analysis using PCA powerfully synthesized the physiological responses of the MAGIC wheat population across diverse environments. The first two principal components efficiently captured the majority of the data’s variability (65.6% to 81.8%). Dimension 1 consistently served as the vigour index, characterized by the high positive loading of NDVI traits and their invariant negative correlation with Canopy Temperature (CT) (Fig 2).

Conversely, Dimension 2 encapsulated the photosynthetic adaptation axis, primarily defined by SPAD chlorophyll content and Fv/Fm. Overall, the PCA results establish NDVI and CT as the foundational traits governing heat tolerance, with SPAD and Fv/Fm providing context-specific insights into the mechanism of adaptation.

### Marker trait associations (MTA) of key physiological traits

**Fig 3:**
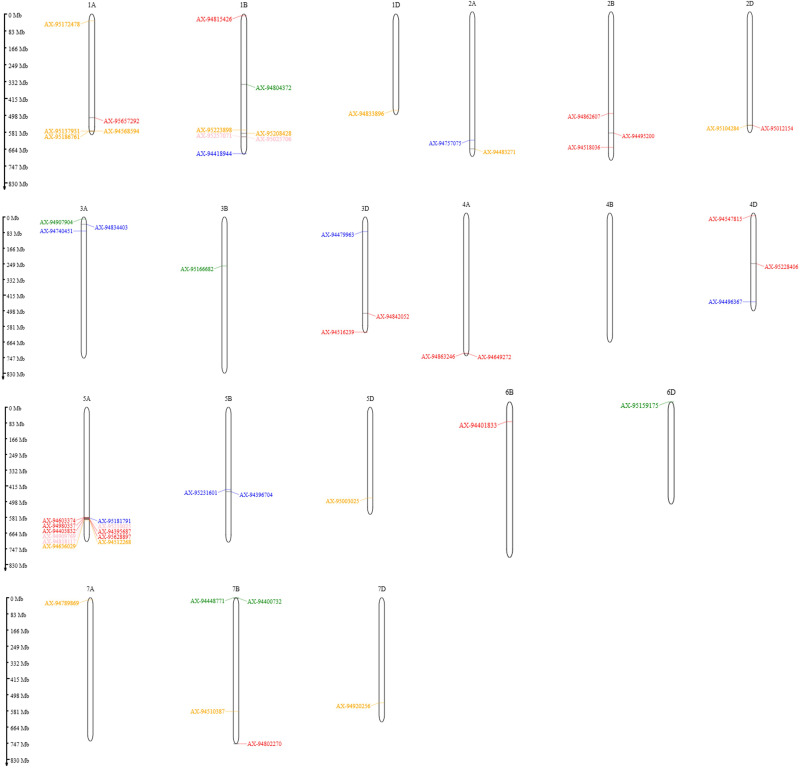
Chromosomal distribution of significant SNPs associated with physiological traits in the MAGIC population. SNPs linked to CT, Fv/Fm, SPAD, and NDVI are represented in blue, green, orange, and red, respectively, while pleiotropic SNPs are shown in pink.

Genome-wide association analysis (GWAS) in the MAGIC wheat population identified 54 significant marker-trait associations (MTA) for key physiological traits- NDVI_1–3, SPAD chlorophyll content, CT, and Fv/Fm UP/LW (upper leaf/lower leaf) under timely and late sown irrigated conditions across multiple environments. Detailed information on these MTA, including their *p*-values and R^2^ values, is provided in S1 Table, while the significant SNPs are illustrated through Manhattan plots (S1 and S2 Fig). The chromosomal distribution of these SNPs is shown in Fig 3.

Major genomic regions were detected on chromosomes 5A, 7A, and 7B, highlighting their consistent contribution to canopy greenness, chlorophyll stability, and photosynthetic efficiency under heat stress. Eighteen MTA were identified for SPAD across both TSIR and LSIR, located on 1A, 1B, 1D, 2A, 2D, 5A, 5D, 7A, 7B, and 7D. A total of 22 MTAs for NDVI were identified across environments, predominantly mapped on chromosome 5A, with additional loci detected on 1A, 1B, 2B, 2D, 3D, 4A, 4D, 6B, and 7B. Eleven MTAs for canopy temperature (CT) were identified, primarily located on chromosomes 5A, 3A, and 5B, with additional loci mapped on 4D, 2A, 3D, and 1B. Eight MTAs for Fv/Fm (upper leaf and lower leaf) were identified, mainly distributed on chromosomes 1B and 7B, with additional loci detected on 6D, 3B, and 3A. Notably, SNPs such as AX-95210025, AX-94980357, and AX-94448771 exhibited stable allelic effects across environments, emphasizing their robustness in conferring heat tolerance. The chromosome 5A region (580–591 Mb) emerged as a key genomic hotspot associated with NDVI and CT and SPAD explaining up to 29.2% of phenotypic variance.

### Allelic effect analysis of significant SNPs

Allelic effect analysis in the MAGIC population identified key SNPs influencing NDVI, SPAD, canopy temperature, and Fv/Fm under both timely and late sown conditions across locations. Favorable alleles, including A (AX-95210025) and G (AX-94403832), consistently increased NDVI by +0.03 to +0.13 (t = 6.72–12.0, P < 0.0001) relative to their alternative allele G; A allele of AX-95210025 and G allele of AX-94496367 significantly reduced canopy temperature by –0.57°C to –1.85°C (t = 6.19, P < 0.0001) compared to their alternative allele G; C alleles of AX-95208428, AX-94789869, and AX-95223898, which increased SPAD (chlorophyll content) values by +5.11 to +7.46 (t = 5.94–6.53, P < 0.0001) while C allele of AX-94448771 and A allele of AX-95159175 improved Fv/Fm (photosynthetic efficiency) by +0.06 (t = 3.17–4.38, P < 0.0001). These resultsindicate improvements in canopy greenness, chlorophyll stability, and heat avoidance (S2 Table). Several loci showed stable, environment-independent effects, reflecting robust genetic control of photosynthetic efficiency. SNPs affecting Fv/Fm confirmed improved PSII performance under heat stress. Co-localization of NDVI and CT loci suggests a shared genetic basis for thermal regulation and photosynthesis.

### In-silico candidate gene analysis

SNP-candidate gene associations underlying key physiological traits in the MAGIC population revealed a complex genetic network governing thermotolerance and photosynthetic stability under heat stress. Genes near significant SNPs were primarily involved in protein stabilization, redox regulation, osmotic balance, and chloroplast protection. Heat shock proteins (e.g., *TraesCS5A02G078000*), osmotin-like, and rubredoxin-like proteins contributed to canopy temperature regulation and maintenance of chlorophyll function (S3 Table). F-box and protein kinase genes enhanced photosystem II efficiency and antioxidant defense, while ubiquitin ligases and phosphatases regulated protein turnover and stress signaling.

### *In silico* expression profiling and GRN analysis of candidate genes associated with physiological traits

**Fig 4:**
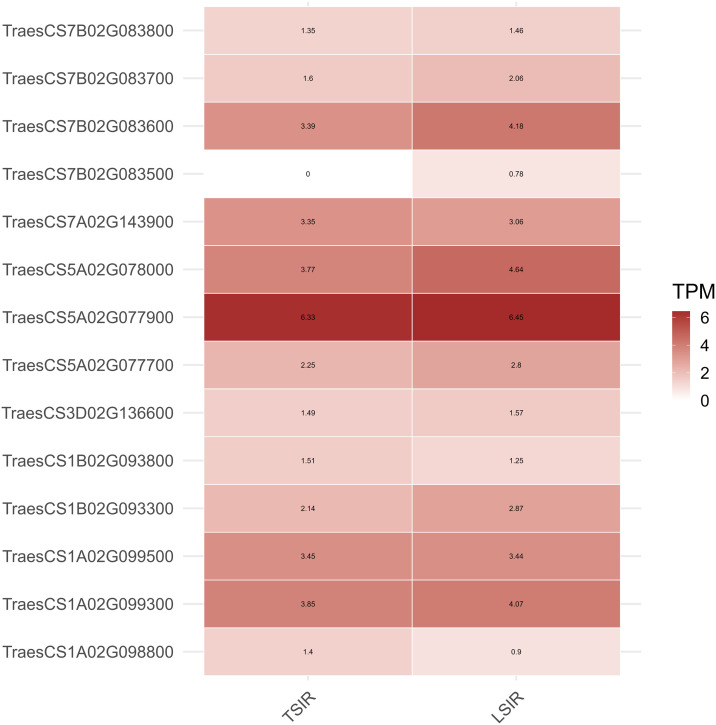
Differential expression of candidate genes associated with key physiological traits in the MAGIC population under Control (TSIR) and Heat Stress (LSIR) Conditions.

The integration of in silico expression profiling and Gene Regulatory Network (GRN) analysis establishes that the seven candidate genes form a cohesive, adaptive system regulating key physiological traits in the MAGIC population under heat stress. Genes associated with SPAD (*TraesCS1A02G098800*, *TraesCS7A02G143900*) show reduced expression consistent with partial chlorophyll degradation, while NDVI genes (*TraesCS1A02G099500*, *TraesCS5A02G077900*/*078000*) maintain or increase expression, supporting canopy greenness by regulating osmotic balance and NPQ to minimize canopy temperature (CT). Crucially, Fv/Fm genes (*TraesCS1B02G093300*, *TraesCS7B02G083500*) are upregulated (Fig 4, S3a-g Fig), reflecting the activation of ABA-driven stress defense and chaperone mechanisms to actively repair Photosystem II, collectively demonstrating a highly synchronized molecular strategy to sustain photosynthetic resilience and yield stability despite thermal challenge.

## Discussion

A comprehensive evaluation of 248 MAGIC population-derived lines and their eight founder parents revealed substantial phenotypic variability and adaptive diversity across environments. Under both timely sown (TSIR) and late sown (LSIR) irrigated conditions, physiological traits NDVI, SPAD chlorophyll content, canopy temperature (CT), and chlorophyll fluorescence (Fv/Fm) exhibited significant genotypic variation, indicating a rich genetic reservoir and strong environmental responsiveness. Genotypes under TSIR conditions showed higher canopy density, chlorophyll content, and photosynthetic efficiency, reflecting superior physiological performance. In contrast, LSIR conditions induced heat stress, characterized by increased CT and reduced SPAD values, highlighting genotypic differences in thermotolerance. Variations in Fv/Fm further indicated differing capacities of photosystem II efficiency and photochemical stability under heat stress. Overall, these results underscore the strong genotype–environment interactions and demonstrate the potential of the MAGIC population for dissecting the genetic and physiological basis of heat tolerance, photosynthetic resilience, and yield stability in wheat.

### Integrated analysis of physiological traits reveals key determinants of heat tolerance in the MAGIC population

Analysis of key physiological traits-NDVI, SPAD (chlorophyll content), canopy temperature (CT), and chlorophyll fluorescence (Fv/Fm) in the MAGIC wheat population revealed consistent interrelationships alongside environment-specific variations. NDVI traits (NDVI_1–3) showed strong inter-correlations across sites (r = 0.71-0.86), reflecting robust canopy vigour, while SPAD (chlorophyll content) correlations were stronger at Delhi (r = 0.62-0.71) but weak or non-significant at Dharwad and Pune, indicating environment-dependent chlorophyll dynamics. Fv/Fm (UP) correlated positively with SPAD chlorophyll content (r = 0.56-0.62) and NDVI_2 (r = 0.52-0.59), while Fv/Fm (LW) showed moderate correlation with Fv/Fm (UP) (r = 0.52). CT consistently exhibited negative correlations with NDVI and Fv/Fm (r = −0.13 to −0.60) (Fig 1), suggesting that cooler canopies correspond to higher greenness and photosynthetic efficiency [26].

PCA supported these findings, with the first two components explaining 65–82% of variance across environments. Dim1 captured the vigour/biomass axis, dominated by positive NDVI and negative CT loadings, highlighting the inverse NDVI-CT relationship under heat stress. Dim2 represented the pigment/stress axis, driven by SPAD (chlorophyll content) and Fv/Fm, reflecting chlorophyll content and photochemical efficiency variation. Across locations, NDVI and CT consistently defined Dim1, while SPAD (chlorophyll content) and Fv/Fm contributions to Dim2 varied (Fig 2), indicating environment-specific adaptive responses [27–30].

### Marker-trait associations for physiological traits in the MAGIC population

Fifty-four significant marker-trait associations (MTA) identified in the MAGIC wheat population for key physiological traits NDVI, SPAD chlorophyll content, canopy temperature (CT), and chlorophyll fluorescence (Fv/Fm) under both timely sown (TSIR) and late sown (LSIR) irrigated conditions across multiple locations (S1 Table). Twenty-two MTA were associated with NDVI measured at three growth stages (NDVI_1–3). Notably, Krishnappa et al. [11] reported MTA for NDVI, showed PVE values ranging from 6.2% to 12.1%, while Gizaw et al. [31] identified loci exhibited PVE values ranging from 3% to 8%. In the present study, major significant SNPs AX-95210025, AX-94403832, AX-94818117, and AX-94980357, explaining up to 29.2% of phenotypic variance, highlighting their major contribution to canopy greenness and photosynthetic vigour under heat stress. Additional MTA for NDVI trait, showed PVE ranging from 2.5-14.3%, and several SNPs (AX-95210025, AX-94980357, AX-94401833, AX-94403832, AX-94547815) showed consistent effects across environments, emphasizing stable genetic control of NDVI (S1Table). Major SNPs identified for SPAD (chlorophyll content) such as AX-94789869 (7A), AX-95104284 (2D), and AX-95208428 (1B) explained 15.1-24.8% of variance, suggesting their role in chlorophyll stability and pigment biosynthesis under heat stress (S1 Table). These findings align with Said et al. [32], who reported SPAD-associated SNPs with PVE ranging from 20.20% to 30.90%. GWAS for CT revealed eleven significant MTA across environments (S1 Table). Major loci AX-95181791, AX-95210025, and AX-94818117 explained up to 22.8% of phenotypic variance, while additional SNPs were indicating multigenic control of thermal response. Chromosome 5A emerged as a key hotspot, consistent with previous reports by Sukumaran et al. [33], linking it to cooler canopy temperatures and improved transpiration efficiency under heat stress. For Fv/Fm, eight SNPs were identified across upper (UP) and lower (LW) leaf (S1 table). For Fv/Fm, Major locus AX-94448771 (7B) explained 13.2% of phenotypic variation of Fv/Fm. Remaining loci accounted for 2.3-6.8%, reflecting cumulative minor effects. These results confirm the polygenic nature of photosynthetic efficiency under heat stress, corroborating findings by Gudi et al. [16], who reported multiple Fv/Fm-associated MTA with substantial effects (>10% PVE). Stable MTA across environments, particularly AX-95210025, AX-94980357, and AX-94547815, identified as robust genetic determinants for these physiological traits

### Allelic effects of significant SNPs across physiological traits in the MAGIC population

Allelic effect analysis in the MAGIC wheat population revealed key SNPs exerting consistent influences on canopy greenness (NDVI), chlorophyll content (SPAD), canopy temperature (CT), and photochemical efficiency (Fv/Fm) across environments (S2 Table). The A allele of AX-95210025 showed strong pleiotropic effects compared to its alternative allele G, enhancing NDVI (+0.03 to +0.13; p < 0.0001) while it reducing canopy temperature (CT) (−0.41°C to −0.57°C, p < 0.0001) along with C allele of AX-95231601, highlighting its role in maintaining canopy vigour, pigment stability, and transpirational cooling under heat stress [31,34]. Similarly, the G allele of AX-94403832 and T allele of AX-94603374 positively influenced NDVI (+0.05 to +0.16, p < 0.0001) and A allele of AX-95025706 and AX-95257071 increased Fv/Fm (photosynthetic efficiency) by +0.01 (P < 0.001) relative to their alternative allele G, indicating improved photosynthetic efficiency and PSII stability [35]. The C alleles of AX-94789869 and AX-95208428 increased SPAD (chlorophyll content) by +5 to +7 units (p < 0.0001) compared to their alternative alleles G and T respectively, reinforcing their contribution to chlorophyll retention and pigment stability [32]. For canopy temperature, favourable alleles such as A allele of AX-94496367 and T AX-95181791 reduced CT (−0.5 to −1.85°C, p < 0.0001) compared to their alternative alleles G and T respectively, reflecting superior thermoregulatory ability [33,36]. Likewise, C allele of AX-94448771 and A allele of AX-95159175 enhanced Fv/Fm by +0.06 (P < 0.01 to p < 0.001) relative to their alternative allele G, supporting sustained PSII performance under stress [16]. Collectively, these loci exhibit additive and pleiotropic effects across multiple physiological traits, underscoring the integrated genetic control of photosynthetic resilience and heat tolerance in wheat.

### Candidate gene identification for key physiological traits

SNPs associated with canopy temperature (CT) were mainly located near genes regulating protein stability, redox homeostasis, and osmotic balance under stress (S3 Table). For instance, AX-95181791 and AX-95210025 are linked to *TraesCS5A02G078000* encoding a Heat shock cognate 70 kDa protein, which stabilizes cellular proteins and membranes to sustain thermotolerance [37], and to *TraesCS5A02G077900* encoding a Rubredoxin-like domain-containing protein that maintains chloroplast stability and prevents photo-oxidative damage [38]. Similarly, AX-94818117 is positioned near *TraesCS5A02G077600* (Osmotin-like protein) that maintains osmotic balance and membrane stability under stress [39]. AX-94396704 maps to *TraesCS5B02G078500* (Protein-serine/threonine phosphatase), which regulates canopy temperature via ROS-mediated signaling [40], whereas AX-94418944 corresponds to *TraesCS1B02G092800* (F-box protein) mediating selective degradation of stress-responsive proteins [41]. Additional SNPs, such as AX-94479963 and AX-94740451, are associated with *TraesCS3D02G135400* (J domain-containing protein/HSP40) and *TraesCS3A02G120700* (Peptidase A1 domain protein), which cooperate in protein refolding and turnover to maintain cellular homeostasis during stress [42,43].

For Maximum quantum yield (Fv/Fm), SNPs associated with this trait, were linked to genes enhancing photosystem II (PSII) stability and repair (S3 Table). For Fv/Fm (LW), AX-94448771 corresponds to *TraesCS7B02G083500* (Small heat shock protein, SHSP domain) that protects PSII from heat-induced denaturation [44], *TraesCS7B02G083600* (RRM domain protein) involved in chloroplast RNA processing for PSII component [45], and *TraesCS7B02G083800* (Serine/threonine-protein phosphatase 2A activator) that regulates PSII phosphorylation cycles [46]. For Fv/Fm (UP), SNPs AX-94907904 and AX-95257071 correspond to *TraesCS3A02G120700* and *TraesCS1B02G093800*, encoding Protein kinases that phosphorylate the D1 protein to protect PSII under heat stress [47]. Additionally, *TraesCS1B02G094000* (F-box protein) and *TraesCS1B02G093300* (G-patch domain protein) contribute to PSII protection via chloroplast RNA stability and stress signaling [45,48].

Significant SNPs influencing NDVI traits were positioned near genes controlling chlorophyll maintenance, protein folding, and antioxidant regulation under stress (S3 Table). For NDVI_1, AX-95210025 is linked to *TraesCS5A02G078000* (Heat shock cognate 70 kDa protein) that protects the photosynthetic apparatus from chlorophyll degradation and to *TraesCS5A02G077600* (Osmotin-like protein) supporting canopy greenness [39]. The same SNP also maps to *TraesCS5A02G077700* (Ubiquitin-related modifier 1 homolog) regulating turnover of photosynthetic proteins [49] and *TraesCS5A02G077900* (Rubredoxin-like protein) maintaining redox balance and chlorophyll stability [50]. AX-95228406 and AX-94980357 are associated with *TraesCS4D02G114200* (HVA22-like protein) and *TraesCS5A02G077900*, both involved in chloroplast protection under stress [50,51]. For NDVI_2 and NDVI_3, key SNPs (AX-94818117, AX-95210025, and AX-95628897) are also linked to *TraesCS5A02G078000* (Heat shock cognate 70 kDa protein) and *TraesCS5A02G077600* (Osmotin-like protein) that sustain chlorophyll biosynthesis and detoxify ROS under heat [52,53]. Furthermore, AX-94862607 and AX-94842052 correspond to *TraesCS2B02G126700* and *TraesCS3D02G136600* (RING-type E3 ubiquitin transferases) regulating chloroplast protein quality and turnover [54], while AX-94863246 maps to *TraesCS4A02G088900* (F-box protein) maintaining photosynthetic capacity under oxidative stress [48].

SNPs of SPAD (chlorophyll content) were predominantly located near genes maintaining chloroplast integrity, redox balance, and photosynthetic efficiency under heat stress (S3 Table). AX-95208428 and AX-95257071 correspond to *TraesCS1B02G093800* (Protein kinase) and *TraesCS1B02G094000* (F-box protein) regulating phosphorylation of light-harvesting proteins and enhancing antioxidant defense [55,56]. AX-95137931 maps to *TraesCS1A02G098800* (DUF4408 domain protein) and *TraesCS1A02G098900* (CCR4-NOT transcription complex subunit 1) maintaining chlorophyll stability via transcriptional and post-transcriptional control of photosynthetic genes [57,58]. Additionally, AX-94789869 is associated with *TraesCS7A02G143900* (Hydroxyproline O-arabinosyltransferase-like protein) ensuring chloroplast membrane stability [59], while AX-94920256 and AX-94568594 correspond to *TraesCS7D02G148000* (Two-component response regulator) and *TraesCS1A02G098100* (HTH myb-type protein), respectively, which modulate chlorophyll metabolism and photosynthetic performance under stress [60,61].

### *In-silico* expression profiling and GRN analysis reveal heat-responsive regulation of photosynthetic efficiency and chlorophyll stability pathways

The in-silico expression profiling of candidate genes associated with physiological traits in the wheat MAGIC population revealed distinct transcriptional responses under TSIR (control) and LSIR (heat stress) conditions. Genes linked to chlorophyll content (SPAD), such as *TraesCS1A02G098800* and *TraesCS7A02G143900*, showed decreased or slightly reduced expression under heat, reflecting chlorophyll degradation and partial pigment stability, respectively [62]. NDVI-associated genes (*TraesCS1A02G099300*, *TraesCS1A02G099500*, *TraesCS3D02G136600*, *TraesCS5A02G077700*) exhibited stable or enhanced expression, suggesting maintained canopy greenness through regulation of photosynthetic and chloroplast maintenance pathways [63]. Notably, *TraesCS5A02G077900* and *TraesCS5A02G078000*, encoding *DnaJ (Hsp40)* and *Heat Shock Cognate 70 kDa (HSC70)* proteins, showed high expression under both conditions, emphasizing their central role in protein stabilization and thermotolerance acquisition, consistent with findings by Poudel et al. [37] and Huang et al. [64]. Genes regulating PSII efficiency (*TraesCS1B02G093300*, *TraesCS7B02G083500*–*083800*) were strongly upregulated under LSIR, indicating activation of chloroplast-localized chaperones that sustain electron transport and photochemical stability under heat (Fig 4). Collectively, the upregulation of HSP–HSC70–DnaJ networks and PSII repair-related genes highlights a coordinated molecular defense that maintains photosynthetic efficiency, canopy greenness, and overall physiological resilience in MAGIC lines under heat stress.

In the MAGIC population, the integrated Gene Regulatory Network (GRN) analysis revealed that all seven candidate genes are intricately connected in regulating the core physiological traits: NDVI, canopy temperature (CT), SPAD chlorophyll content, and Fv/Fm. *TraesCS1A02G098800* acts as a pleiotropic hub coordinating chlorophyll biosynthesis and photosynthetic efficiency, directly enhancing SPAD and indirectly influencing NDVI, Fv/Fm, and CT through its impact on pigment stability and metabolic regulation [65]. *TraesCS1A02G099500* regulates NDVI and Fv/Fm by controlling photosynthetic greenness, biomass accumulation, and stress-responsive transcriptional networks, thereby maintaining CT and chlorophyll stability under heat stress. *TraesCS1B02G093300* links NDVI and Fv/Fm through modulation of light energy utilization and oxidative stress defense, ensuring sustained photosystem function and chlorophyll fluorescence efficiency. *TraesCS5A02G077900* and *TraesCS5A02G078000* integrate NDVI, CT, and SPAD by enhancing non-photochemical quenching (NPQ), osmotic balance, and hormone-mediated stress tolerance [66], collectively supporting cooler canopy temperature and stable green tissue. Meanwhile, *TraesCS7A02G143900* connects light signaling with chlorophyll metabolism to stabilize SPAD and optimize photosynthetic capacity, while *TraesCS7B02G083500* safeguards Fv/Fm and NDVI through ABA-driven stress adaptation and photosystem II repair mechanisms (S3a-g Fig). Together, these genes form a cohesive regulatory framework in the MAGIC population that synchronizes chlorophyll retention, canopy greenness, photochemical efficiency, and thermal regulation key determinants of photosynthetic resilience and yield stability under heat stress.

## Conclusion

The study demonstrates substantial genetic and physiological diversity within the MAGIC wheat population, reflecting strong genotype–environment interactions and adaptive potential under heat stress. NDVI and CT were key indicators of canopy vigor and thermoregulation, while SPAD and Fv/Fm represented chlorophyll stability and photosynthetic efficiency. GWAS identified 54 stable MTA- predominantly on chromosome 5A, with major loci (AX-95210025, AX-94980357, AX-94448771) showing pleiotropic effects on canopy greenness, chlorophyll content, and PSII stability. Candidate gene and GRN analyses revealed a coordinated molecular network involving *TraesCS1A02G099500* (controlling photosynthetic greenness), *TraesCS1A02G098800* (chlorophyll biosynthesis), *TraesCS5A02G077900* and *TraesCS5A02G078000* (heat shock proteins mediating protein stability and canopy cooling), and *TraesCS7B02G083500* (PSII protection). Together with *TraesCS1B02G093300* and *TraesCS7A02G143900*, these genes regulate chlorophyll retention, redox balance, and photochemical resilience. Collectively, these integrated genetic and regulatory insights identify key alleles and hub genes for marker-assisted selection and genomic breeding of heat-tolerant, photosynthetically efficient wheat cultivars.

## Supporting information

S1 Table Significant marker–trait associations for physiological traits different environmental conditions in MAGIC Population. (DOCX)

S2 Table Allelic effects of significant SNPs associated with physiological traits in the MAGIC population under heat stress. (DOCX)

S3 Table Putative candidate genes identified within ±100 kb region of linked SNPs, along with their molecular functions. (DOCX)

S4 Table Meteorological data (maximum, minimum, and mean temperatures) recorded at the time of physiological trait measurements during the 2024−25 *Rabi* season at Delhi under TSIR and LSIR conditions. (DOCX)

S5 Table Meteorological data (maximum, minimum, and mean temperatures) recorded at the time of physiological trait measurements during the 2024−25 *Rabi* season at Dharwad under TSIR and LSIR conditions. (DOCX)

S6 Table Meteorological data (maximum, minimum, and mean temperatures) recorded at the time of physiological trait measurements during the 2024−25 *Rabi* season at Pune under TSIR and LSIR conditions. (DOCX)

S7 Table Meteorological data for the 2024−25 Rabi season at the Delhi station. (DOCX)

S8 Table Meteorological data for the 2024−25 Rabi season at the Dharwad station. (DOCX)

S9 Table Meteorological data for the 2024−25 Rabi season at the Pune station. (DOCX)

S1 Fig Manhattan plots depicting SNP associations for physiological traits under TSIR condition across location during the 2024−25 season. (DOCX)

S2 Fig Manhattan plots depicting SNP associations for physiological traits under LSIR condition across location during the 2024−25 season. (DOCX)

S3 Fig Gene regulatory networks of candidate genes associated with physiological traits in the MAGIC population. *TraesCS1A02G098800*(**a**), *TraesCS1A02G099500*(**b**), *TraesCS1B02G093300*(**c**), *TraesCS5A02G077900*(**d**), *TraesCS5A02G078000*(**e**) *TraesCS7A02G143900* (**f**) and *TraesCS7B02G083500* (**g**).
(DOCX)
