# Supplementary material for: Genetic mapping and candidate gene identification for key physiological traits associated with heat tolerance in wheat (Triticum aestivum L.) using a MAGIC population
Source: PLoS One. 2026 Jan 2;21(1):e0339966. doi: 10.1371/journal.pone.0339966 (PMC12758712; doi:10.1371/journal.pone.0339966)
Supplement: S1 Table — (DOCX) [file pone.0339966.s001.docx]

**S1 Table. Significant marker-trait associations for physiological traits different environmental conditions in MAGIC Population**

| **Traits** | **Env.** | **SNP** | **Chr. No.** | **Pos. in Mb** | **P.value** | **R2. Squared** | **-log_10_(p)** |
| --- | --- | --- | --- | --- | --- | --- | --- |
| NDVI_1 | LS_DHAR | AX-95210025 | 5A | 585.4129 | 9.39E-12 | 18.8 | 11.03 |
| NDVI_1 | LS_DHAR | AX-94403832 | 5A | 588.7411 | 5.18E-05 | 14.7 | 4.286 |
| NDVI_1 | LS_DL | AX-95012154 | 2D | 611.1609 | 0.000334 | 3.3 | 3.502 |
| NDVI_1 | LS_DL | AX-94518036 | 2B | 730.6137 | 0.000195 | 2.87 | 3.711 |
| NDVI_1 | LS_PUNE | AX-94980357 | 5A | 585.0709 | 2.58E-08 | 7.77 | 7.588 |
| NDVI_1 | LS_PUNE | AX-94815426 | 1B | 9.598542 | 0.000217 | 3.76 | 3.664 |
| NDVI_1 | LS_PUNE | AX-95657292 | 1A | 508.3208 | 0.000216 | 2.62 | 3.665 |
| NDVI_1 | TS_DHAR | AX-94403832 | 5A | 588.7411 | 5.3E-07 | 14.6 | 6.276 |
| NDVI_1 | TS_DHAR | AX-94395687 | 5A | 588.8722 | 1.3E-06 | 10.9 | 5.887 |
| NDVI_1 | TS_PUNE | AX-95210025 | 5A | 585.4129 | 8.45E-17 | 15.9 | 16.07 |
| NDVI_1 | TS_PUNE | AX-95228406 | 4D | 262.6518 | 0.000177 | 9.51 | 3.752 |
| NDVI_2 | LS_DHAR | AX-94980357 | 5A | 585.0709 | 1.19E-07 | 25.3 | 6.925 |
| NDVI_2 | LS_DL | AX-94802270 | 7B | 749.4081 | 0.000127 | 8.28 | 3.896 |
| NDVI_2 | LS_DL | AX-94547815 | 4D | 14.98635 | 0.000251 | 2.54 | 3.601 |
| NDVI_2 | LS_PUNE | AX-95210025 | 5A | 585.4129 | 4.8E-18 | 20.1 | 17.32 |
| NDVI_2 | TS_DHAR | AX-94818117 | 5A | 591.4635 | 4.35E-24 | 24.6 | 23.36 |
| NDVI_2 | TS_DHAR | AX-94516239 | 3D | 613.4019 | 8.35E-07 | 9.13 | 6.079 |
| NDVI_2 | TS_DHAR | AX-94547815 | 4D | 14.98635 | 0.000198 | 2.96 | 3.702 |
| NDVI_2 | TS_DL | AX-94495200 | 2B | 653.5949 | 0.000283 | 4.44 | 3.547 |
| NDVI_2 | TS_PUNE | AX-95210025 | 5A | 585.4129 | 2.56E-19 | 22.1 | 18.59 |
| NDVI_2 | TS_PUNE | AX-94401833 | 6B | 92.56933 | 7.64E-06 | 11.6 | 5.117 |
| NDVI_3 | LS_DHAR | AX-95210025 | 5A | 585.4129 | 3.72E-25 | 25 | 24.43 |
| NDVI_3 | LS_DHAR | AX-94842052 | 3D | 512.2718 | 8.94E-07 | 8.08 | 6.049 |
| NDVI_3 | LS_PUNE | AX-95210025 | 5A | 585.4129 | 5.23E-22 | 28.4 | 21.28 |
| NDVI_3 | TS_DHAR | AX-95210025 | 5A | 585.4129 | 1.42E-26 | 29.2 | 25.85 |
| NDVI_3 | TS_DHAR | AX-95628897 | 5A | 591.3185 | 0.000319 | 14.3 | 3.497 |
| NDVI_3 | TS_DHAR | AX-94401833 | 6B | 92.56933 | 0.000189 | 12.8 | 3.723 |
| NDVI_3 | TS_DHAR | AX-94649272 | 4A | 733.574 | 0.000331 | 3.53 | 3.5 |
| NDVI_3 | TS_DHAR | AX-94863246 | 4A | 732.5124 | 0.000282 | 3.17 | 3.549 |
| NDVI_3 | TS_DL | AX-94980357 | 5A | 585.0709 | 0.00023 | 13.4 | 3.638 |
| NDVI_3 | TS_DL | AX-94909769 | 5A | 589.5853 | 0.000169 | 12.6 | 3.771 |
| NDVI_3 | TS_PUNE | AX-94403832 | 5A | 588.7411 | 6.01E-06 | 15.2 | 5.221 |
| NDVI_3 | TS_PUNE | AX-94603374 | 5A | 580.803 | 5.45E-05 | 13.7 | 4.263 |
| NDVI_3 | TS_PUNE | AX-94862607 | 2B | 549.3085 | 0.00018 | 9.11 | 3.745 |
| SPAD | LS_DHAR | AX-94833896 | 1D | 472.5277 | 0.000303 | 4.68 | 3.519 |
| SPAD | LS_DHAR | AX-94512268 | 5A | 591.4874 | 3.05E-05 | 4.51 | 4.516 |
| SPAD | LS_DHAR | AX-95003025 | 5D | 477.8324 | 0.000148 | 3.72 | 3.829 |
| SPAD | LS_DHAR | AX-94636029 | 5A | 596.4473 | 0.000113 | 3.67 | 3.946 |
| SPAD | LS_DL | AX-94789869 | 7A | 9.940279 | 0.000297 | 24.8 | 3.527 |
| SPAD | LS_DL | AX-95208428 | 1B | 586.4513 | 0.000178 | 15.1 | 3.751 |
| SPAD | LS_DL | AX-95223898 | 1B | 570.842 | 0.0001 | 6.27 | 4 |
| SPAD | LS_DL | AX-95172478 | 1A | 32.61515 | 0.000259 | 5.51 | 3.586 |
| SPAD | LS_DL | AX-95257071 | 1B | 603.951 | 0.000124 | 3.55 | 3.906 |
| SPAD | LS_DL | AX-95025706 | 1B | 603.9557 | 0.000145 | 2.68 | 3.839 |
| SPAD | TS_DL | AX-95104284 | 2D | 611.1606 | 0.00017 | 17.8 | 3.77 |
| SPAD | TS_DL | AX-95025706 | 1B | 603.9557 | 6.58E-05 | 9.28 | 4.182 |
| SPAD | TS_DL | AX-95257071 | 1B | 603.951 | 8.98E-05 | 9.12 | 4.047 |
| SPAD | TS_DL | AX-94483271 | 2A | 739.2766 | 0.000279 | 8.85 | 3.554 |
| SPAD | TS_PUNE | AX-95137931 | 1A | 576.3733 | 0.00029 | 5.27 | 3.538 |
| SPAD | TS_PUNE | AX-94920256 | 7D | 541.3358 | 1.42E-05 | 5.05 | 4.846 |
| SPAD | TS_PUNE | AX-94510387 | 7B | 585.3088 | 0.000322 | 4.8 | 3.493 |
| SPAD | TS_PUNE | AX-94909769 | 5A | 589.5853 | 0.000186 | 4.59 | 3.73 |
| SPAD | TS_PUNE | AX-95186761 | 1A | 576.674 | 4.38E-05 | 3.09 | 4.359 |
| SPAD | TS_PUNE | AX-94568594 | 1A | 576.674 | 0.0002 | 3.03 | 3.699 |
| CT | LS_DHAR | AX-95181791 | 5A | 584.673 | 5.89E-17 | 22.8 | 16.23 |
| CT | LS_DHAR | AX-94834403 | 3A | 39.30811 | 0.000325 | 2.65 | 3.5 |
| CT | LS_DL | AX-94496367 | 4D | 462.7444 | 6.24E-07 | 7.67 | 6.205 |
| CT | LS_DL | AX-94740451 | 3A | 74.78305 | 2.60E-04 | 2.78 | 3.585 |
| CT | LS_PUNE | AX-94757075 | 2A | 692.85 | 0.000305 | 5.7 | 3.516 |
| CT | LS_PUNE | AX-94396704 | 5B | 444.7513 | 2.42E-05 | 5.39 | 4.616 |
| CT | LS_PUNE | AX-94479963 | 3D | 76.92749 | 0.000221 | 3.27 | 3.656 |
| CT | LS_PUNE | AX-95231601 | 5B | 435.4458 | 3.22E-04 | 3.25 | 3.5 |
| CT | TS_DHAR | AX-94818117 | 5A | 591.4635 | 2.28E-11 | 10.7 | 10.64 |
| CT | TS_PUNE | AX-95210025 | 5A | 585.4129 | 1.87E-09 | 13.4 | 8.727 |
| CT | TS_PUNE | AX-94418944 | 1B | 686.4753 | 0.000337 | 3.87 | 3.5 |
| Fv/Fm LW | LS_DL | AX-95159175 | 6D | 4.41E-06 | 0.000166 | 2.75 | 3.78 |
| Fv/Fm LW | TS_DL | AX-94448771 | 7B | 0.000684 | 0.000254 | 13.2 | 3.6 |
| Fv/Fm UP | LS_DL | AX-95166682 | 3B | 259.4693 | 0.000106 | 3.44 | 3.975 |
| Fv/Fm UP | LS_DL | AX-94400732 | 7B | 2.928607 | 0.000326 | 2.31 | 3.5 |
| Fv/Fm UP | TS_DL | AX-94907904 | 3A | 11.40986 | 0.000158 | 6.82 | 3.802 |
| Fv/Fm UP | TS_DL | AX-95257071 | 1B | 603.951 | 0.000153 | 6.02 | 3.815 |
| Fv/Fm UP | TS_DL | AX-95025706 | 1B | 603.9557 | 0.000114 | 5.88 | 3.942 |
| Fv/Fm UP | TS_DL | AX-94804372 | 1B | 347.1058 | 0.000249 | 5.36 | 3.603 |

TS, Timely sown irrigated condition (TSIR); LS, Late sown irrigated condition (LSIR); DL, Delhi; DHAR, Dharwad; PUNE, Pune; Env., Environment; SNP, Single nucleotide polymorphism; Chr. No., Chromosome number; Pos. in Mb, Physical position in million base pairs; R²: Percentage of phenotypic variance explained; –log₁₀(p), Negative logarithm of p-value indicating the level of marker significance. Significant marker–trait associations (MTAs) were identified based on a threshold of p < 0.001.
