# Supplementary material for: Genetic mapping and candidate gene identification for key physiological traits associated with heat tolerance in wheat (Triticum aestivum L.) using a MAGIC population
Source: PLoS One. 2026 Jan 2;21(1):e0339966. doi: 10.1371/journal.pone.0339966 (PMC12758712; doi:10.1371/journal.pone.0339966)
Supplement: S2 Table — (DOCX) [file pone.0339966.s002.docx]

**S2 Table. Allelic effects of significant SNPs associated with physiological traits in the MAGIC population under heat stress.**

| **Traits** | **Conditions** | **SNPs** | **Alleles** | **Mean ± SD** | **Allele effect** | **t stat** | **t critical** | **P value** | **Significance ^a^** |
| --- | --- | --- | --- | --- | --- | --- | --- | --- | --- |
| Fv/Fm LW | TS_DL | AX-94448771 | C | 0.64±0.03 | 0.06 | 4.38 | 1.8 | 0.000553 | *** |
|  |  |  | G | 0.58±0.05 |  |  |  |  |  |
| Fv/Fm LW | LS_DL | AX-95159175 | A | 0.6±0.05 | 0.06 | 3.17 | 1.74 | 0.00277 | ** |
|  |  |  | G | 0.54±0.07 |  |  |  |  |  |
| Fv/Fm UP | TS_DL | AX-95257071 | A | 0.7±0.02 | 0.01 | 3.31 | 1.65 | 0.000546 | *** |
|  |  |  | G | 0.71±0.01 |  |  |  |  |  |
| Fv/Fm UP | TS_DL | AX-95025706 | A | 0.7±0.02 | 0.01 | 3.37 | 1.65 | 0.00045 | *** |
|  |  |  | G | 0.71±0.01 |  |  |  |  |  |
| Fv/Fm UP | LS_DL | AX-95166682 | C | 0.68±0.02 | 0.01 | 1.7 | 1.66 | 0.04622 | * |
|  |  |  | T | 0.67±0.02 |  |  |  |  |  |
| Fv/ Fm UP | LS_DL | AX-94400732 | G | 0.68±0.01 | 0.01 | 1.9 | 1.66 | 0.030168 | * |
|  |  |  | T | 0.67±0.02 |  |  |  |  |  |
| NDVI_1 | TS_DHAR | AX-94403832 | A | 0.7±0.04 | 0.04 | 8.11 | 1.66 | 8.38E-13 | *** |
|  |  |  | G | 0.74±0.03 |  |  |  |  |  |
| NDVI_1 | TS_DHAR | AX-94395687 | A | 0.71±0.04 | 0.04 | 6.78 | 1.68 | 8.8E-09 | *** |
|  |  |  | G | 0.75±0.03 |  |  |  |  |  |
| NDVI_1 | LS_DHAR | AX-95210025 | A | 0.76±0.02 | 0.03 | 9.82 | 1.66 | 5.16E-18 | *** |
|  |  |  | G | 0.73±0.02 |  |  |  |  |  |
| NDVI_1 | LS_DHAR | AX-94403832 | A | 0.73±0.02 | 0.03 | 7.97 | 1.66 | 2.49E-12 | *** |
|  |  |  | G | 0.76±0.02 |  |  |  |  |  |
| NDVI_1 | TS_PUNE | AX-95210025 | A | 0.88±0.02 | 0.03 | 9.38 | 1.65 | 1.56E-17 | *** |
|  |  |  | G | 0.85±0.02 |  |  |  |  |  |
| NDVI_1 | TS_PUNE | AX-95228406 | C | 0.85±0.02 | 0.01 | 4.1 | 1.66 | 3.45E-05 | *** |
|  |  |  | T | 0.87±0.02 |  |  |  |  |  |
| NDVI_1 | LS_DL | AX-94980357 | A | 0.8±0.01 | 0.01 | 4.61 | 1.66 | 5.94E-06 | *** |
|  |  |  | C | 0.81±0.01 |  |  |  |  |  |
| NDVI_2 | TS_DL | AX-94495200 | A | 0.53±0.05 | 0.04 | 5.76 | 1.65 | 2.17E-08 | *** |
|  |  |  | G | 0.49±0.06 |  |  |  |  |  |
| NDVI_2 | LS_DL | AX-94802270 | A | 0.54±0.05 | 0.08 | 5.45 | 1.76 | 4.31E-05 | *** |
|  |  |  | C | 0.46±0.05 |  |  |  |  |  |
| NDVI_2 | LS_DL | AX-94547815 | A | 0.54±0.05 | 0.04 | 4.16 | 1.68 | 6.15E-05 | *** |
|  |  |  | G | 0.5±0.05 |  |  |  |  |  |
| NDVI_2 | TS_DHAR | AX-94818117 | C | 0.6±0.04 | 0.05 | 9.76 | 1.66 | 5.09E-18 | *** |
|  |  |  | T | 0.65±0.04 |  |  |  |  |  |
| NDVI_2 | TS_DHAR | AX-94516239 | A | 0.61±0.04 | 0.03 | 4.02 | 1.66 | 5.28E-05 | *** |
|  |  |  | G | 0.64±0.04 |  |  |  |  |  |
| NDVI_2 | TS_DHAR | AX-94547815 | A | 0.62±0.04 | 0.03 | 2.81 | 1.68 | 0.003657 | ** |
|  |  |  | G | 0.6±0.05 |  |  |  |  |  |
| NDVI_2 | LS_DHAR | AX-94980357 | A | 0.65±0.04 | 0.06 | 8.8 | 1.66 | 8.39E-14 | *** |
|  |  |  | C | 0.71±0.05 |  |  |  |  |  |
| NDVI_2 | TS_PUNE | AX-95210025 | A | 0.77±0.04 | 0.06 | 9.84 | 1.65 | 1.71E-18 | *** |
|  |  |  | G | 0.71±0.04 |  |  |  |  |  |
| NDVI_2 | TS_PUNE | AX-94401833 | A | 0.71±0.04 | 0.05 | 4.66 | 1.67 | 7.65E-06 | *** |
|  |  |  | G | 0.76±0.05 |  |  |  |  |  |
| NDVI_2 | LS_PUNE | AX-95210025 | A | 0.69±0.03 | 0.04 | 8.91 | 1.65 | 5.37E-16 | *** |
|  |  |  | G | 0.65±0.03 |  |  |  |  |  |
| NDVI_3 | TS_DL | AX-94980357 | A | 0.29±0.04 | 0.04 | 5.63 | 1.66 | 1.04E-07 | *** |
|  |  |  | C | 0.33±0.05 |  |  |  |  |  |
| NDVI_3 | TS_DHAR | AX-95210025 | G | 0.3±0.07 | 0.13 | 12 | 1.65 | 4.66E-24 | *** |
|  |  |  | A | 0.42±0.07 |  |  |  |  |  |
| NDVI_3 | TS_DHAR | AX-94401833 | A | 0.3±0.08 | 0.1 | 4.75 | 1.67 | 5.29E-06 | *** |
|  |  |  | G | 0.4±0.09 |  |  |  |  |  |
| NDVI_3 | TS_DHAR | AX-94649272 | A | 0.33±0.09 | 0.05 | 2.96 | 1.67 | 0.002061 | ** |
|  |  |  | T | 0.38±0.09 |  |  |  |  |  |
| NDVI_3 | LS_DHAR | AX-95210025 | A | 0.37±0.09 | 0.11 | 10.2 | 1.66 | 2.86E-18 | *** |
|  |  |  | G | 0.25±0.06 |  |  |  |  |  |
| NDVI_3 | LS_DHAR | AX-94842052 | C | 0.28±0.09 | 0.07 | 4.44 | 1.67 | 2.22E-05 | *** |
|  |  |  | T | 0.35±0.08 |  |  |  |  |  |
| NDVI_3 | TS_PUNE | AX-94403832 | A | 0.5±0.11 | 0.13 | 6.74 | 1.67 | 1.33E-09 | *** |
|  |  |  | G | 0.63±0.11 |  |  |  |  |  |
| NDVI_3 | TS_PUNE | AX-94603374 | C | 0.68±0.09 | 0.16 | 7.06 | 1.71 | 1.07E-07 | *** |
|  |  |  | T | 0.51±0.12 |  |  |  |  |  |
| NDVI_3 | TS_PUNE | AX-94862607 | C | 0.5±0.11 | 0.08 | 4.19 | 1.65 | 2.24E-05 | *** |
|  |  |  | T | 0.58±0.13 |  |  |  |  |  |
| NDVI_3 | LS_PUNE | AX-95210025 | A | 0.58±0.06 | 0.1 | 11.3 | 1.65 | 1.32E-22 | *** |
|  |  |  | G | 0.48±0.07 |  |  |  |  |  |
| CT | LS_DL | AX-94496367 | A | 30.4±2.05 | 1.85 | 6 | 1.66 | 1.23E-08 | *** |
|  |  |  | G | 32.3±2 |  |  |  |  |  |
| CT | TS_DHAR | AX-94818117 | C | 24.4±0.59 | 0.5 | 6.07 | 1.65 | 4.31E-09 | *** |
|  |  |  | T | 23.9±0.57 |  |  |  |  |  |
| CT | LS_DHAR | AX-95181791 | G | 25.6±0.44 | 0.59 | 7.25 | 1.66 | 1.01E-10 | *** |
|  |  |  | T | 25±0.51 |  |  |  |  |  |
| CT | LS_DHAR | AX-94834403 | C | 25.2±0.56 | 0.37 | 4.43 | 1.67 | 1.93E-05 | *** |
|  |  |  | T | 25.6±0.4 |  |  |  |  |  |
| CT | TS_PUNE | AX-95210025 | G | 27.9±0.69 | 0.57 | 6.19 | 1.65 | 2.07E-09 | *** |
|  |  |  | A | 27.4±0.6 |  |  |  |  |  |
| CT | LS_PUNE | AX-94396704 | C | 29.1±0.75 | 0.44 | 4.16 | 1.66 | 3.41E-05 | *** |
|  |  |  | T | 29.6±0.59 |  |  |  |  |  |
| CT | LS_PUNE | AX-94479963 | G | 29.4±0.67 | 0.46 | 3.93 | 1.68 | 0.000164 | *** |
|  |  |  | T | 29.8±0.56 |  |  |  |  |  |
| CT | LS_PUNE | AX-95231601 | C | 29.5±0.61 | 0.41 | 3.65 | 1.67 | 0.000241 | *** |
|  |  |  | T | 29.1±0.72 |  |  |  |  |  |
| SPAD | TS_DL | AX-95025706 | A | 38.2±10.9 | 4.4 | 3.01 | 1.65 | 0.001469 | ** |
|  |  |  | G | 42.6±8.66 |  |  |  |  |  |
| SPAD | TS_DL | AX-95257071 | A | 38.6±10.8 | 4.6 | 2.91 | 1.65 | 0.001992 | ** |
|  |  |  | G | 43.2±8.52 |  |  |  |  |  |
| SPAD | LS_DL | AX-94789869 | C | 46±4.45 | 5.63 | 6.53 | 1.66 | 8.22E-10 | *** |
|  |  |  | G | 40.4±6.66 |  |  |  |  |  |
| SPAD | LS_DL | AX-95208428 | C | 44.6±6.04 | 7.46 | 6.44 | 1.67 | 6.83E-09 | *** |
|  |  |  | T | 37.2±6.91 |  |  |  |  |  |
| SPAD | LS_DL | AX-95223898 | C | 44.9±5.51 | 5.11 | 4.94 | 1.66 | 1.75E-06 | *** |
|  |  |  | G | 39.8±7.22 |  |  |  |  |  |
| SPAD | LS_DHAR | AX-94512268 | A | 48.3±1.82 | 1.19 | 3.79 | 1.67 | 0.000162 | *** |
|  |  |  | G | 47.1±1.86 |  |  |  |  |  |
| SPAD | LS_DHAR | AX-94636029 | A | 48.3±1.81 | 1.14 | 3.66 | 1.67 | 0.000253 | *** |
|  |  |  | C | 47.2±1.85 |  |  |  |  |  |
| SPAD | TS_PUNE | AX-95137931 | C | 48.2±1.09 | 0.71 | 2.98 | 1.68 | 0.002289 | ** |
|  |  |  | T | 47.4±1.29 |  |  |  |  |  |
| SPAD | TS_PUNE | AX-95186761 | C | 47.5±1.37 | 0.67 | 3.2 | 1.66 | 0.000977 | *** |
|  |  |  | T | 48.2±1.09 |  |  |  |  |  |
| SPAD | TS_PUNE | AX-94568594 | C | 48.2±1.09 | 0.57 | 3.26 | 1.66 | 0.00069 | *** |
|  |  |  | T | 47.6±1.31 |  |  |  |  |  |

TS, timely sown irrigated condition (TSIR); LS, late sown irrigated condition (LSIR); DL, Delhi; DHAR, Dharwad; PUNE, Pune; SNP, single nucleotide polymorphism; SD, standard deviation; t stat, t statistic; t critical, critical t value; P value, probability value.

^a^ Significance levels: p < 0.05 (), p < 0.01 (), p < 0.001
