# Supplementary material for: Genetic mapping and candidate gene identification for key physiological traits associated with heat tolerance in wheat (Triticum aestivum L.) using a MAGIC population
Source: PLoS One. 2026 Jan 2;21(1):e0339966. doi: 10.1371/journal.pone.0339966 (PMC12758712; doi:10.1371/journal.pone.0339966)
Supplement: S3 Table — (DOCX) [file pone.0339966.s003.docx]

**S3 Table. Putative candidate genes identified** **within ±100 kb region of linked SNPs, along with their molecular functions.**

| **Traits** | **SNPs** | **Chr. No.** | **Gene ID** | **Proteins** | **Functions** | **References** |
| --- | --- | --- | --- | --- | --- | --- |
| CT | AX-95181791 | 5A | TraesCS5A02G077900 | Rubredoxin-like domain-containing protein | Maintains chloroplast stability and prevents photo-oxidative damages under stress. | Ostria-Gallardo et al., [38] |
| CT | AX-95181791 | 5A | TraesCS5A02G078000 | Heat shock cognate 70 kDa protein | Stabilize cellular proteins and membranes under heat stress. | Poudel et al., [37] |
| CT | AX-95210025 | 5A | TraesCS5A02G078000 | Heat shock cognate 70 kDa protein | Stabilize cellular proteins and membranes under heat stress. | Poudel et al., [37] |
| CT | AX-95210025 | 5A | TraesCS5A02G077900 | Rubredoxin-like domain containing protein | Maintains chloroplast stability and prevents photo-oxidative damage. | Ostria-Gallardo et al., [38] |
| CT | AX-94818117 | 5A | TraesCS5A02G077600 | Osmotin-like protein | Maintains cellular osmotic balance and membrane stability. | Bashir et al., [39] |
| CT | AX-94818117 | 5A | TraesCS5A02G077900 | Rubredoxin-like domain-containing protein | Maintains chloroplast stability and prevents photo-oxidative damage. | Ostria-Gallardo et al., [38] |
| CT | AX-94396704 | 5B | TraesCS5B02G078500 | protein-serine/threonine phosphatase | Regulates canopy temperature by modulating ROS-mediated signaling and antioxidant responses. | Mathe et al., [40] |
| CT | AX-94418944 | 1B | TraesCS1B02G092800 | F-box domain-containing protein | Mediating selective degradation of stress-responsive proteins. | Guerin et al., [41] |
| CT | AX-94479963 | 3D | TraesCS3D02G135300 | Glycosyltransferases | Stabilizing secondary metabolites and hormones, enhancing antioxidant defense. | Malakondaiah et al., [15] |
| CT | AX-94479963 | 3D | TraesCS3D02G135400 | J domain-containing protein/HSP 40 | Cooperating with Hsp70 chaperones to prevent protein misfolding and aggregation. | Arif et al., [42] |
| CT | AX-94740451 | 3A | TraesCS3A02G120700 | Peptidase A1 domain-containing protein | Facilitates controlled protein degradation and turnover during stress-induced senescence. | Velasco Arroyo et al., [43] |
| Fv/Fm LW | AX-94448771 | 7B | TraesCS7B02G083500 | SHSP domain-containing protein | Maintain Photosystem II stability by protecting its proteins from stress-induced denaturation and aggregation. | Kumar et al., [44] |
| Fv/Fm LW | AX-94448771 | 7B | TraesCS7B02G083600 | RRM domain-containing protein | Regulate PSII indirectly by binding and processing chloroplast RNAs that encode PSII components. | Wang et al., [45] |
| Fv/Fm LW | AX-94448771 | 7B | TraesCS7B02G083700 | F-box domain-containing protein | Maintain photosystem II efficiency (Fv/Fm) by protecting the photosynthetic machinery. | Li et al., [48] |
| Fv/Fm LW | AX-94448771 | 7B | TraesCS7B02G083800 | Serine/threonine-protein phosphatase 2A activator | Regulates phosphorylation dephosphorylation of PSII proteins. | Naraikina et al., [46] |
| Fv/Fm UP | AX-94907904 | 3A | TraesCS3A02G120700 | Protein kinase domain-containing protein | Regulates PSII by phosphorylating the D1 protein, protecting it from stress-induced degradation. | Zhao et al., [47] |
| Fv/Fm UP | AX-95257071 | 1B | TraesCS1B02G094000 | F-box domain-containing protein | Protects the photosynthetic machinery and enhancing stress-responsive pathways. | Li et al., [48] |
| Fv/Fm UP | AX-95257071 | 1B | TraesCS1B02G093800 | Protein kinase domain-containing protein | Regulates PSII by phosphorylating the D1 protein, protecting it under stress. | Zhao et al., [47] |
| Fv/Fm UP | AX-94804372 | 1B | TraesCS1B02G093300 | D11/G-patch domain-containing protein | Proper maturation and stability of chloroplast RNAs that encode PSII components | Wang et al., [45] |
| Fv/Fm UP | AX-94400732 | 7B | TraesCS7B02G083700 | F-box domain-containing protein | Protects the photosynthetic machinery and enhancing stress-responsive pathways. | Li et al., [48] |
| NDVI_1 | AX-95210025 | 5A | TraesCS5A02G078000 | Heat shock cognate 70 kDa protein | Protects the photosynthetic apparatus and preventing chlorophyll degradation. | Poudel et al., [37] |
| NDVI_1 | AX-95210025 | 5A | TraesCS5A02G077600 | Osmotin-like protein | Sustains photosynthetic activity and canopy greenness. | Basir et al., [39] |
| NDVI_1 | AX-95210025 | 5A | TraesCS5A02G077700 | Ubiquitin-related modifier 1 homolog | Leaf greenness by regulating the stability and turnover of photosynthetic proteins. | Sun et al., [49] |
| NDVI_1 | AX-95210025 | 5A | TraesCS5A02G077900 | Rubredoxin-like domain-containing protein | Maintains redox balance and protecting chlorophyll. | Vukadinović et al., [50] |
| NDVI_1 | AX-95228406 | 4D | TraesCS4D02G114200 | HVA22-like protein | Protects chloroplasts, and maintaining chlorophyll under stress. | Zhang et al., [51] |
| NDVI_1 | AX-94980357 | 5A | TraesCS5A02G077900 | Rubredoxin-like domain-containing protein | Maintaining redox balance and supporting chlorophyll function, | Vukadinović et al., [50] |
| NDVI_1 | AX-94815426 | 1B | TraesCS1B02G093300 | G-patch domain-containing protein | Supporting optimal gene expression for photosynthetic activity, | Wang et al., [45] |
| NDVI_1 | AX-95657292 | 1A | TraesCS1A02G099300 | DnaJ protein ERDJ3B | Proper protein folding and chloroplast development under stress. | Arif et al., [42] |
| NDVI_2 | AX-94818117 | 5A | TraesCS5A02G077600 | Osmotin-like protein | Regulates osmotic balance and ROS detoxification. | Chowdhury et al., [53] |
| NDVI_2 | AX-95210025 | 5A | TraesCS5A02G078000 | Heat shock cognate 70 kDa protein | Chlorophyll biosynthesis and stability under stress by preventing protein denaturation | Aghaie et al., [52] |
| NDVI_2 | AX-94516239 | 3D | TraesCS3D02G135400 | J domain-containing protein | Co-chaperone with HSP70 to facilitate proper protein folding and preserving chlorophyll biosynthesis. | Arif et al., [42] |
| NDVI_3 | AX-95210025 | 5A | TraesCS5A02G078000 | Heat shock cognate 70 kDa protein | Prevents protein denaturation and supporting chloroplast function, thereby sustaining photosynthetic efficiency | Aghaie et al., [52] |
| NDVI_3 | AX-95210025 | 5A | TraesCS5A02G077600 | Osmotin-like protein | Regulates osmotic balance and ROS detoxification, thereby maintaining chlorophyll stability | Chowdhury et al., [53] |
| NDVI_3 | AX-95628897 | 5A | TraesCS5A02G078000 | Heat shock cognate 70 kDa protein | Prevents protein denaturation and supporting chloroplast function, thereby sustaining photosynthetic efficiency | Aghaie et al., [52] |
| NDVI_3 | AX-94980357 | 5A | TraesCS5A02G077900 | Rubredoxin-like domain-containing protein | Maintaining redox balance and protecting chlorophyll, thus sustaining canopy greenness and NDVI. | Vukadinović et al., [50] |
| NDVI_3 | AX-94862607 | 2B | TraesCS2B02G126700 | RING-type E3 ubiquitin transferase | Regulates chloroplast protein quality and turnover under stress, maintaining photosynthetic efficiency and chlorophyll stability | Hand et al., [54] |
| NDVI_3 | AX-94842052 | 3D | TraesCS3D02G136600 | RING-type domain-containing protein | Regulates chloroplast protein quality and turnover under stress, maintaining photosynthetic efficiency and chlorophyll stability | Hand et al., [54] |
| NDVI_3 | AX-94863246 | 4A | TraesCS4A02G088900 | F-box domain-containing protein | Maintaining photosynthetic capacity and reducing oxidative damag | Li et al., [48] |
| SPAD | AX-94789869 | 7A | TraesCS7A02G143900 | Hydroxyproline O-arabinosyltransferase-like domain-containing protein | Maintains chloroplast membrane stability, indirectly protecting chlorophyll and supporting photosynthesis under stress. | Singh et al., [59] |
| SPAD | AX-95208428 | 1B | TraesCS1B02G093800 | Protein kinase domain-containing protein | Regulates chloroplast development and chlorophyll function by modulating phosphorylation of light-harvesting proteins and balancing energy distribution between PSII and PSI. | Baker et al., [55] |
| SPAD | AX-95257071 | 1B | TraesCS1B02G094000 | F-box domain-containing protein | Protects chlorophyll and maintain chloroplast function under stress by enhancing antioxidant defense. | Zhou et al., [56] |
| SPAD | AX-95137931 | 1A | TraesCS1A02G098800 | DUF4408 domain-containing protein | Regulate chloroplast development and maintain chlorophyll function. | Luo et al., [57] |
| SPAD | AX-95137931 | 1A | TraesCS1A02G098900 | CCR4-NOT transcription complex subunit 1 CAF1-binding domain-containing protein | Regulates chloroplast function by mediating mRNA decay of photosynthetic genes. | Su et al., [58] |
| SPAD | AX-94920256 | 7D | TraesCS7D02G148000 | Two-component response regulator | Modulates chlorophyll metabolism and chloroplast function. | He et al., [60] |
| SPAD | AX-94568594 | 1A | TraesCS1A02G097900 | NADH-ubiquinone oxidoreductase chain 6 | Maintaining chlorophyll content and fluorescence stability. | Gudi et al., [16] |
| SPAD | AX-94568594 | 1A | TraesCS1A02G098100 | HTH myb-type domain-containing protein | Maintain chlorophyll stability and photosynthetic efficiency under heat stress. | Ma et al., [61] |

CT, canopy temperature; Fv/Fm, chlorophyll fluorescence; NDVI, normalized difference vegetation index; SPAD, SPAD chlorophyll content; SNP, single nucleotide polymorphism; Chr. No., chromosome number; Gene ID, Ensembl gene identifier of *Triticum aestivum*.
