# Supplementary material for: Genetic mapping and candidate gene identification for key physiological traits associated with heat tolerance in wheat (Triticum aestivum L.) using a MAGIC population
Source: PLoS One. 2026 Jan 2;21(1):e0339966. doi: 10.1371/journal.pone.0339966 (PMC12758712; doi:10.1371/journal.pone.0339966)
Supplement: S4 Table — (DOCX) [file pone.0339966.s004.docx]

**S4 Table. Meteorological data (maximum, minimum, and mean temperatures) recorded at the time of physiological trait measurements during the 2024-25 *Rabi* season at Delhi under TSIR and LSIR conditions.**

| Traits | Env. | Date measured | Max. Temp (°C) | Min. Temp (°C) | Mean Temp (°C) |
| --- | --- | --- | --- | --- | --- |
| NDVI 1 | TS_DL | 02.02.2025 | 22.2 | 8.0 | 15.1 |
| NDVI 1 | LS_DL | 01.03.2025 | 25.0 | 17.0 | 21 |
| NDVI 2 | TS_DL | 25.02.2025 | 23.3 | 8.2 | 15.75 |
| NDVI 2 | LS_DL | 22.03.2025 | 32.2 | 14.6 | 23.4 |
| NDVI 3 | TS_DL | 13.03.2025 | 34.6 | 18.6 | 26.6 |
| NDVI 3 | LS_DL | 03.04.2025 | 35.7 | 13.9 | 24.8 |
| CT 1 | TS_DL | 17.02.2025 | 29.2 | 10.4 | 19.8 |
| CT 1 | LS_DL | 16.03.2025 | 32.5 | 16.0 | 24.25 |
| CT 2 | TS_DL | 27.02.2025 | 31.1 | 19.1 | 25.1 |
| CT 2 | LS_DL | 25.03.2025 | 36.0 | 14.1 | 25.05 |
| SPAD 1 | TS_DL | 03.02.2025 | 23.0 | 9.2 | 16.1 |
| SPAD 1 | LS_DL | 04.03.2025 | 30.8 | 12.7 | 21.75 |
| SPAD 2 | TS_DL | 13.02.2025 | 25.2 | 12.4 | 18.8 |
| SPAD 2 | LS_DL | 14.03.2025 | 33.8 | 18.0 | 25.9 |
| Fv/Fm LW | TS_DL | 18.02.2025 | 28.6 | 12.7 | 20.65 |
| Fv/Fm LW | LS_DL | 17.03.2025 | 31.3 | 16.6 | 23.95 |
| Fv/Fm UP | TS_DL | 18.02.2025 | 28.6 | 12.7 | 20.65 |
| Fv/Fm UP | LS_DL | 17.03.2025 | 31.3 | 16.6 | 23.95 |

TS, timely sown irrigated condition (TSIR); LS, late sown irrigated condition (LSIR); DL, Delhi
