# Supplementary material for: Genetic mapping and candidate gene identification for key physiological traits associated with heat tolerance in wheat (Triticum aestivum L.) using a MAGIC population
Source: PLoS One. 2026 Jan 2;21(1):e0339966. doi: 10.1371/journal.pone.0339966 (PMC12758712; doi:10.1371/journal.pone.0339966)
Supplement: S5 Table — (DOCX) [file pone.0339966.s005.docx]

**S5 Table. Meteorological data (maximum, minimum, and mean temperatures) recorded at the time of physiological trait measurements during the 2024-25 *Rabi* season at Dharwad under TSIR and LSIR conditions.**

| Traits | Env. | Date measured | Max. Temp (°C) | Min. Temp (°C) | Mean Temp (°C) |
| --- | --- | --- | --- | --- | --- |
| NDVI 1 | TS_DHAR | 20.12.2024 | 29.2 | 16.4 | 22.8 |
| NDVI 1 | LS_DHAR | 02.01.2025 | 28.8 | 13.6 | 21.2 |
| NDVI 2 | TS_DHAR | 12.01.2025 | 29.2 | 16.6 | 22.9 |
| NDVI 2 | LS_DHAR | 25.01.2025 | 32.8 | 11.6 | 22.2 |
| NDVI 3 | TS_DHAR | 01.02.2025 | 33.2 | 15.2 | 24.2 |
| NDVI 3 | LS_DHAR | 20.02.2025 | 34 | 19 | 26.5 |
| CT 1 | TS_DHAR | 02.01.2025 | 28.8 | 13.6 | 21.2 |
| CT 1 | LS_DHAR | 12.01.2025 | 29.2 | 16.6 | 22.9 |
| CT 2 | TS_DHAR | 12.01.2025 | 29.2 | 16.6 | 22.9 |
| CT 2 | LS_DHAR | 25.01.2025 | 32.8 | 11.6 | 22.2 |
| SPAD 1 | TS_DHAR | 20.12.2024 | 29.2 | 16.4 | 22.8 |
| SPAD 1 | LS_DHAR | 02.01.2025 | 28.8 | 13.6 | 21.2 |
| SPAD 2 | TS_DHAR | 12.01.2025 | 29.2 | 16.6 | 22.9 |
| SPAD 2 | LS_DHAR | 25.01.2025 | 32.8 | 11.6 | 22.2 |

TS, timely sown irrigated condition (TSIR); LS, late sown irrigated condition (LSIR); DHAR, Dharwad
