# Supplementary material for: Genetic mapping and candidate gene identification for key physiological traits associated with heat tolerance in wheat (Triticum aestivum L.) using a MAGIC population
Source: PLoS One. 2026 Jan 2;21(1):e0339966. doi: 10.1371/journal.pone.0339966 (PMC12758712; doi:10.1371/journal.pone.0339966)
Supplement: S6 Table — (DOCX) [file pone.0339966.s006.docx]

**S6 Table. Meteorological data (maximum, minimum, and mean temperatures) recorded at the time of physiological trait measurements during the 2024-25 *Rabi* season at Pune under TSIR and LSIR conditions.**

| Traits | Env. | Date measured | Max. Temp (°C) | Min. Temp (°C) | Mean Temp (°C) |
| --- | --- | --- | --- | --- | --- |
| NDVI 1 | TS_PUNE | 09.01.2025 | 30.7 | 10.5 | 20.6 |
| NDVI 1 | LS_PUNE | 24.01.2025 | 35.5 | 13 | 24.25 |
| NDVI 2 | TS_PUNE | 04.02.2025 | 34.2 | 13.4 | 23.8 |
| NDVI 2 | LS_PUNE | 20.02.2025 | 36 | 15.2 | 25.6 |
| NDVI 3 | TS_PUNE | 19.02.2025 | 34.7 | 14.8 | 24.75 |
| NDVI 3 | LS_PUNE | 01.03.2025 | 35 | 15.2 | 25.1 |
| CT 1 | TS_PUNE | 25.01.2025 | 33.8 | 13.5 | 23.65 |
| CT 1 | LS_PUNE | 05.02.2025 | 34.2 | 13.4 | 23.8 |
| CT 2 | TS_PUNE | 05.02.2025 | 34.2 | 13.4 | 23.8 |
| CT 2 | LS_PUNE | 15.02.2025 | 34.2 | 12 | 23.1 |
| SPAD 1 | TS_PUNE | 10.01.2025 | 31 | 10.5 | 20.75 |
| SPAD 1 | LS_PUNE | 23.01.2025 | 32.8 | 12.4 | 22.6 |
| SPAD 2 | TS_PUNE | 25.01.25 | 33.8 | 13.5 | 23.65 |
| SPAD 2 | LS_PUNE | 06.02.2025 | 34.5 | 14.9 | 24.7 |

TS, timely sown irrigated condition (TSIR); LS, late sown irrigated condition (LSIR); PUNE, Pune
