# Supplementary material for: Genetic mapping and candidate gene identification for key physiological traits associated with heat tolerance in wheat (Triticum aestivum L.) using a MAGIC population
Source: PLoS One. 2026 Jan 2;21(1):e0339966. doi: 10.1371/journal.pone.0339966 (PMC12758712; doi:10.1371/journal.pone.0339966)
Supplement: S7 Table — (DOCX) [file pone.0339966.s007.docx]

**S7 Table.** **Meteorological data for the 2024-25 Rabi season at the Delhi station.**

| Date | Max.Temp. (°C) | Min.Temp. (°C) | RH Max (%) | RH Min (%) | Rainfall (mm) |
| --- | --- | --- | --- | --- | --- |
| 01-Nov-24 | 33.4 | 17.1 | 79.0 | 36.0 | 0.0 |
| 02-Nov-24 | 33.7 | 15.6 | 81.0 | 50.0 | 0.0 |
| 03-Nov-24 | 32.0 | 16.6 | 83.0 | 43.0 | 0.0 |
| 04-Nov-24 | 31.9 | 15.3 | 94.0 | 46.0 | 0.0 |
| 05-Nov-24 | 32.0 | 16.3 | 92.0 | 55.0 | 0.0 |
| 06-Nov-24 | 31.8 | 15.9 | 94.0 | 49.0 | 0.0 |
| 07-Nov-24 | 32.1 | 17.1 | 92.0 | 50.0 | 0.0 |
| 08-Nov-24 | 32.1 | 17.3 | 92.0 | 59.0 | 0.0 |
| 09-Nov-24 | 31.3 | 17.8 | 87.0 | 53.0 | 0.0 |
| 10-Nov-24 | 31.6 | 18.4 | 90.0 | 48.0 | 0.0 |
| 11-Nov-24 | 318.0 | 17.5 | 86.0 | 47.0 | 0.0 |
| 12-Nov-24 | 32.5 | 15.4 | 71.0 | 29.0 | 0.0 |
| 13-Nov-24 | 32.7 | 18.4 | 94.0 | 62.0 | 0.0 |
| 14-Nov-24 | 27.7 | 14.8 | 94.0 | 55.0 | 0.0 |
| 15-Nov-24 | 29.0 | 14.5 | 93.0 | 46.0 | 0.0 |
| 16-Nov-24 | 29.6 | 14.0 | 88.0 | 50.0 | 0.0 |
| 17-Nov-24 | 29.5 | 13.7 | 91.0 | 54.0 | 0.0 |
| 18-Nov-24 | 27.4 | 14.5 | 87.0 | 62.0 | 0.0 |
| 19-Nov-24 | 22.8 | 10.8 | 88.0 | 49.0 | 0.0 |
| 20-Nov-24 | 24.9 | 9.4 | 86.0 | 50.0 | 0.0 |
| 21-Nov-24 | 25.6 | 8.7 | 87.0 | 43.0 | 0.0 |
| 22-Nov-24 | 26.0 | 9.8 | 91.0 | 47.0 | 0.0 |
| 23-Nov-24 | 26.6 | 10.0 | 90.0 | 43.0 | 0.0 |
| 24-Nov-24 | 30.0 | 11.2 | 88.0 | 43.0 | 0.0 |
| 25-Nov-24 | 28.2 | 11.8 | 84.0 | 56.0 | 0.0 |
| 26-Nov-24 | 25.5 | 9.8 | 86.0 | 55.0 | 0.0 |
| 27-Nov-24 | 26.3 | 9.2 | 92.0 | 38.0 | 0.0 |
| 28-Nov-24 | 26.6 | 8.0 | 94.0 | 33.0 | 0.0 |
| 29-Nov-24 | 26.2 | 7.9 | 89.0 | 44.0 | 0.0 |
| 30-Nov-24 | 25.8 | 8.0 | 92.0 | 41.0 | 0.0 |
| 01-Dec-24 | 25.4 | 8.2 | 88.0 | 46.0 | 0.0 |
| 02-Dec-24 | 24.5 | 8.6 | 90.0 | 41.0 | 0.0 |
| 03-Dec-24 | 26.6 | 7.8 | 89.0 | 41.0 | 0.0 |
| 04-Dec-24 | 27.8 | 13.5 | 67.0 | 28.0 | 0.0 |
| 05-Dec-24 | 27.5 | 7.5 | 79.0 | 32.0 | 0.0 |
| 06-Dec-24 | 24.5 | 6.6 | 84.0 | 33.0 | 0.0 |
| 07-Dec-24 | 24.2 | 5.6 | 81.0 | 35.0 | 0.0 |
| 08-Dec-24 | 24.6 | 6.0 | 87.0 | 39.0 | 0.0 |
| 09-Dec-24 | 22.2 | 5.7 | 84.0 | 47.0 | 0.0 |
| 10-Dec-24 | 21.5 | 6.9 | 84.0 | 34.0 | 0.0 |
| 11-Dec-24 | 22.2 | 6.1 | 67.0 | 31.0 | 0.0 |
| 12-Dec-24 | 22.3 | 1.6 | 80.0 | 32.0 | 0.0 |
| 13-Dec-24 | 21.9 | 9.1 | 64.0 | 40.0 | 0.0 |
| 14-Dec-24 | 22.2 | 4.8 | 78.0 | 30.0 | 0.0 |
| 15-Dec-24 | 22.9 | 2.4 | 83.0 | 44.0 | 0.0 |
| 16-Dec-24 | 21.6 | 1.8 | 76.0 | 50.0 | 0.0 |
| 17-Dec-24 | 23.5 | 4.4 | 88.0 | 47.0 | 0.0 |
| 18-Dec-24 | 21.8 | 3.6 | 94.0 | 55.0 | 0.0 |
| 19-Dec-24 | 21.2 | 3.5 | 87.0 | 62.0 | 0.0 |
| 20-Dec-24 | 21.7 | 5.8 | 83.0 | 69.0 | 0.0 |
| 21-Dec-24 | 22.4 | 5.6 | 86.0 | 67.0 | 0.0 |
| 22-Dec-24 | 22.4 | 5.2 | 83.0 | 66.0 | 0.0 |
| 23-Dec-24 | 22.0 | 12.0 | 97.0 | 73.0 | 0.2 |
| 24-Dec-24 | 18.0 | 12.3 | 93.0 | 74.0 | 0.2 |
| 25-Dec-24 | 20.0 | 7.4 | 92.0 | 71.0 | 0.2 |
| 26-Dec-24 | 21.2 | 6.1 | 89.0 | 68.0 | 0.0 |
| 27-Dec-24 | 22.8 | 11.5 | 97.0 | 98.0 | 9.0 |
| 28-Dec-24 | 14.8 | 11.8 | 93.0 | 91.0 | 40.1 |
| 29-Dec-24 | 15.7 | 12.9 | 95.0 | 93.0 | 0.0 |
| 30-Dec-24 | 16.0 | 8.9 | 92.0 | 88.0 | 0.0 |
| 31-Dec-24 | 16.2 | 8.3 | 92.0 | 80.0 | 0.0 |
| 01-Jan-25 | 17.8 | 6.8 | 95.0 | 84.0 | 0.0 |
| 02-Jan-25 | 15.2 | 7.0 | 94.0 | 84.0 | 0.0 |
| 03-Jan-25 | 15.4 | 5.9 | 95.0 | 84.0 | 0.0 |
| 04-Jan-25 | 18.2 | 6.5 | 89.0 | 82.0 | 0.0 |
| 05-Jan-25 | 19.4 | 5.8 | 94.0 | 84.0 | 0.0 |
| 06-Jan-25 | 17.4 | 8.2 | 95.0 | 87.0 | 0.4 |
| 07-Jan-25 | 15.3 | 8.0 | 90.0 | 87.0 | 0.0 |
| 08-Jan-25 | 14.4 | 6.2 | 88.0 | 71.0 | 0.0 |
| 09-Jan-25 | 20.2 | 5.1 | 85.0 | 77.0 | 0.0 |
| 10-Jan-25 | 21.3 | 4.9 | 97.0 | 83.0 | 0.0 |
| 11-Jan-25 | 17.1 | 6.7 | 95.0 | 87.0 | 0.0 |
| 12-Jan-25 | 16.6 | 9.2 | 95.0 | 81.0 | 2.3 |
| 13-Jan-25 | 16.3 | 10.1 | 95.0 | 76.0 | 0.0 |
| 14-Jan-25 | 17.8 | 8.7 | 90.0 | 82.0 | 0.0 |
| 15-Jan-25 | 17.6 | 4.5 | 92.0 | 76.0 | 0.0 |
| 16-Jan-25 | 17.0 | 9.4 | 95.0 | 80.0 | 8.6 |
| 17-Jan-25 | 18.2 | 8.5 | 92.0 | 77.0 | 0.0 |
| 18-Jan-25 | 16.0 | 10.1 | 93.0 | 69.0 | 0.0 |
| 19-Jan-25 | 18.2 | 8.9 | 90.0 | 59.0 | 0.0 |
| 20-Jan-25 | 25.0 | 11.1 | 86.0 | 64.0 | 0.0 |
| 21-Jan-25 | 23.3 | 10.8 | 84.0 | 61.0 | 0.0 |
| 22-Jan-25 | 23.3 | 7.9 | 87.0 | 52.0 | 0.0 |
| 23-Jan-25 | 24.4 | 7.9 | 95.0 | 65.0 | 0.0 |
| 24-Jan-25 | 23.8 | 10.2 | 86.0 | 71.0 | 0.0 |
| 25-Jan-25 | 23.3 | 8.2 | 89.0 | 67.0 | 0.0 |
| 26-Jan-25 | 22.2 | 4.8 | 88.0 | 70.0 | 0.0 |
| 27-Jan-25 | 21.4 | 5.6 | 86.0 | 62.0 | 0.0 |
| 28-Jan-25 | 22.4 | 4.1 | 94.0 | 64.0 | 0.0 |
| 29-Jan-25 | 23.9 | 6.0 | 86.0 | 70.0 | 0.0 |
| 30-Jan-25 | 23.9 | 10.0 | 92.0 | 58.0 | 0.0 |
| 31-Jan-25 | 26.1 | 8.4 | 95.0 | 68.0 | 0.0 |
| 01-Feb-25 | 25.9 | 9.6 | 90.0 | 70.0 | 0.0 |
| 02-Feb-25 | 22.2 | 8.0 | 87.0 | 62.0 | 0.0 |
| 03-Feb-25 | 23.0 | 9.2 | 92.0 | 63.0 | 0.0 |
| 04-Feb-25 | 22.9 | 13.1 | 84.0 | 81.0 | 0.2 |
| 05-Feb-25 | 25.3 | 10.6 | 97.0 | 58.0 | 0.0 |
| 06-Feb-25 | 23.0 | 9.5 | 79.0 | 55.0 | 0.0 |
| 07-Feb-25 | 22.1 | 10.6 | 77.0 | 57.0 | 0.0 |
| 08-Feb-25 | 23.3 | 9.0 | 79.0 | 48.0 | 0.0 |
| 09-Feb-25 | 24.9 | 9.8 | 81.0 | 51.0 | 0.0 |
| 10-Feb-25 | 27.2 | 7.9 | 83.0 | 63.0 | 0.0 |
| 11-Feb-25 | 27.0 | 8.6 | 78.0 | 57.0 | 0.0 |
| 12-Feb-25 | 28.5 | 9.9 | 82.0 | 48.0 | 0.0 |
| 13-Feb-25 | 25.2 | 12.4 | 80.0 | 35.0 | 0.0 |
| 14-Feb-25 | 24.9 | 10.6 | 71.0 | 53.0 | 0.0 |
| 15-Feb-25 | 25.5 | 11.0 | 76.0 | 54.0 | 0.0 |
| 16-Feb-25 | 26.8 | 12.0 | 71.0 | 54.0 | 0.0 |
| 17-Feb-25 | 29.2 | 10.4 | 86.0 | 57.0 | 0.0 |
| 18-Feb-25 | 28.6 | 12.7 | 95.0 | 59.0 | 0.0 |
| 19-Feb-25 | 27.9 | 9.1 | 77.0 | 64.0 | 0.0 |
| 20-Feb-25 | 28.9 | 14.8 | 93.0 | 71.0 | 1.0 |
| 21-Feb-25 | 25.8 | 12.1 | 82.0 | 67.0 | 0.0 |
| 22-Feb-25 | 26.0 | 13.0 | 88.0 | 73.0 | 0.0 |
| 23-Feb-25 | 24.0 | 10.1 | 84.0 | 72.0 | 0.0 |
| 24-Feb-25 | 26.8 | 9.1 | 77.0 | 70.0 | 0.0 |
| 25-Feb-25 | 26.7 | 10.5 | 78.0 | 65.0 | 0.0 |
| 26-Feb-25 | 29.1 | 14.6 | 86.0 | 71.0 | 0.0 |
| 27-Feb-25 | 31.1 | 19.1 | 83.0 | 81.0 | 0.0 |
| 28-Feb-25 | 25.6 | 18.5 | 87.0 | 74.0 | 0.0 |
| 01-Mar-25 | 25.0 | 17.0 | 88.0 | 66.0 | 2.2 |
| 02-Mar-25 | 27.1 | 14.6 | 80.0 | 61.0 | 0.0 |
| 03-Mar-25 | 28.0 | 10.6 | 82.0 | 61.0 | 0.0 |
| 04-Mar-25 | 30.8 | 12.7 | 76.0 | 48.0 | 0.0 |
| 05-Mar-25 | 31.2 | 12.1 | 65.0 | 29.0 | 0.0 |
| 06-Mar-25 | 24.8 | 12.0 | 74.0 | 48.0 | 0.0 |
| 07-Mar-25 | 28.1 | 7.6 | 75.0 | 67.0 | 0.0 |
| 08-Mar-25 | 28.5 | 10.4 | 72.0 | 68.0 | 0.0 |
| 09-Mar-25 | 30.9 | 10.1 | 87.0 | 67.0 | 0.0 |
| 10-Mar-25 | 31.7 | 17.9 | 81.0 | 79.0 | 0.0 |
| 11-Mar-25 | 31.2 | 14.9 | 84.0 | 67.0 | 0.0 |
| 12-Mar-25 | 34.2 | 16.3 | 90.0 | 56.0 | 0.0 |
| 13-Mar-25 | 34.6 | 18.6 | 89.0 | 62.0 | 0.0 |
| 14-Mar-25 | 33.8 | 18.0 | 85.0 | 54.0 | 0.0 |
| 15-Mar-25 | 35.2 | 16.9 | 78.0 | 56.0 | 0.0 |
| 16-Mar-25 | 32.5 | 16.0 | 81.0 | 61.0 | 0.0 |
| 17-Mar-25 | 31.3 | 16.6 | 70.0 | 65.0 | 0.0 |
| 18-Mar-25 | 29.0 | 13.9 | 82.0 | 65.0 | 0.0 |
| 19-Mar-25 | 30.0 | 13.2 | 83.0 | 57.0 | 0.0 |
| 20-Mar-25 | 32.8 | 13.5 | 85.0 | 57.0 | 0.0 |
| 21-Mar-25 | 33.8 | 15.4 | 80.0 | 49.0 | 0.0 |
| 22-Mar-25 | 32.2 | 14.6 | 79.0 | 61.0 | 0.0 |
| 23-Mar-25 | 31.6 | 13.5 | 84.0 | 62.0 | 0.0 |
| 24-Mar-25 | 33.6 | 13.1 | 83.0 | 53.0 | 0.0 |
| 25-Mar-25 | 36.0 | 14.1 | 79.0 | 52.0 | 0.0 |
| 26-Mar-25 | 37.2 | 15.1 | 79.0 | 49.0 | 0.0 |
| 27-Mar-25 | 39.0 | 17.6 | 82.0 | 49.0 | 0.0 |
| 28-Mar-25 | 36.4 | 19.9 | 57.0 | 48.0 | 0.0 |
| 29-Mar-25 | 32.2 | 15.6 | 46.0 | 40.0 | 0.0 |
| 30-Mar-25 | 33.0 | 15.2 | 62.0 | 38.0 | 0.0 |
| 31-Mar-25 | 34.4 | 14.5 | 71.0 | 31.0 | 0.0 |
| 01-Apr-25 | 34.8 | 16.6 | 76.0 | 43.0 | 0.0 |
| 02-Apr-25 | 34.2 | 12.5 | 56.0 | 44.0 | 0.0 |
| 03-Apr-25 | 35.7 | 13.9 | 68.0 | 46.0 | 0.0 |
| 04-Apr-25 | 38.2 | 16.5 | 75.0 | 47.0 | 0.0 |
| 05-Apr-25 | 37.8 | 18.4 | 73.0 | 42.0 | 0.0 |
| 06-Apr-25 | 38.2 | 19.8 | 66.0 | 47.0 | 0.0 |
| 07-Apr-25 | 38.5 | 18.6 | 70.0 | 53.0 | 0.0 |
| 08-Apr-25 | 40.4 | 23.0 | 51.0 | 45.0 | 0.0 |
| 09-Apr-25 | 39.3 | 24.7 | 75.0 | 43.0 | 0.0 |
| 10-Apr-25 | 39.9 | 24.6 | 65.0 | 49.0 | 0.0 |
| 11-Apr-25 | 40.2 | 22.5 | 74.0 | 65.0 | 0.0 |
| 12-Apr-25 | 34.2 | 19.4 | 81.0 | 55.0 | 0.2 |
| 13-Apr-25 | 35.5 | 21.1 | 78.0 | 60.0 | 0.0 |
| 14-Apr-25 | 36.2 | 21.4 | 73.0 | 53.0 | 0.0 |
| 15-Apr-25 | 36.0 | 23.4 | 73.0 | 51.0 | 0.0 |
| 16-Apr-25 | 36.8 | 22.4 | 68.0 | 52.0 | 0.0 |
| 17-Apr-25 | 38.9 | 25.1 | 57.0 | 47.0 | 0.0 |
| 18-Apr-25 | 40.8 | 24.6 | 73.0 | 46.0 | 0.0 |
| 19-Apr-25 | 39.6 | 24.2 | 68.0 | 45.0 | 0.2 |
| 20-Apr-25 | 38.4 | 23.9 | 64.0 | 42.0 | 0.0 |
| 21-Apr-25 | 40.0 | 25.8 | 68.0 | 40.0 | 0.0 |
| 22-Apr-25 | 41.2 | 20.0 | 54.0 | 36.0 | 0.0 |
| 23-Apr-25 | 40.4 | 18.5 | 51.0 | 32.0 | 0.0 |
| 24-Apr-25 | 39.5 | 18.2 | 59.0 | 43.0 | 0.0 |
| 25-Apr-25 | 41.0 | 19.6 | 51.0 | 33.0 | 0.0 |
| 26-Apr-25 | 41.8 | 18.7 | 63.0 | 35.0 | 0.0 |
| 27-Apr-25 | 42.6 | 24.5 | 50.0 | 40.0 | 0.0 |
| 28-Apr-25 | 41.4 | 23.4 | 44.0 | 40.0 | 0.0 |
| 29-Apr-25 | 39.1 | 23.0 | 60.0 | 54.0 | 0.0 |
| 30-Apr-25 | 36.1 | 25.3 | 62.0 | 57.0 | 0.0 |
