# Supplementary material for: Genetic mapping and candidate gene identification for key physiological traits associated with heat tolerance in wheat (Triticum aestivum L.) using a MAGIC population
Source: PLoS One. 2026 Jan 2;21(1):e0339966. doi: 10.1371/journal.pone.0339966 (PMC12758712; doi:10.1371/journal.pone.0339966)
Supplement: S8 Table — (DOCX) [file pone.0339966.s008.docx]

**S8 Table.** **Meteorological data for the 2024-25 Rabi season at the Dharwad station.**

| Date | Max.Temp (°C) | Min.Temp (°C) | RH Max (%) | RH Min (%) | Rainfall (mm) | NRD (days) |
| --- | --- | --- | --- | --- | --- | --- |
| 01-Nov-24 | 31.6 | 20 | 91 | 61 | 0.8 | 0 |
| 02-Nov-24 | 31.6 | 20 | 74 | 79 | 0 | 0 |
| 03-Nov-24 | 31 | 19.4 | 84 | 17 | 0 | 0 |
| 04-Nov-24 | 31 | 19.5 | 75 | 53 | 0 | 0 |
| 05-Nov-24 | 30.4 | 18 | 69 | 50 | 0 | 0 |
| 06-Nov-24 | 30.6 | 15.6 | 62 | 43 | 0 | 0 |
| 07-Nov-24 | 30.8 | 15.4 | 60 | 61 | 0 | 0 |
| 08-Nov-24 | 30.6 | 15.4 | 60 | 41 | 0 | 0 |
| 09-Nov-24 | 31.2 | 16.8 | 74 | 50 | 0 | 0 |
| 10-Nov-24 | 30.4 | 17 | 77 | 51 | 0 | 0 |
| 11-Nov-24 | 30.4 | 16.5 | 62 | 44 | 0 | 0 |
| 12-Nov-24 | 29.8 | 15.8 | 63 | 39 | 0 | 0 |
| 13-Nov-24 | 30 | 19.2 | 77 | 43 | 0 | 0 |
| 14-Nov-24 | 30.2 | 19.6 | 79 | 53 | 0 | 0 |
| 15-Nov-24 | 30.6 | 20.4 | 88 | 56 | 0 | 0 |
| 16-Nov-24 | 28.8 | 19.2 | 85 | 64 | 0 | 0 |
| 17-Nov-24 | 30.4 | 17 | 82 | 48 | 0 | 0 |
| 18-Nov-24 | 29.8 | 16.5 | 68 | 16 | 0 | 0 |
| 19-Nov-24 | 29 | 15.8 | 78 | 44 | 0 | 0 |
| 20-Nov-24 | 28.8 | 13 | 66 | 42 | 0 | 0 |
| 21-Nov-24 | 28.6 | 13.8 | 66 | 43 | 0 | 0 |
| 22-Nov-24 | 29.8 | 14 | 71 | 44 | 0 | 0 |
| 23-Nov-24 | 29.6 | 13 | 75 | 70 | 0 | 0 |
| 24-Nov-24 | 29.6 | 13.5 | 65 | 36 | 0 | 0 |
| 25-Nov-24 | 29.6 | 13 | 70 | 33 | 0 | 0 |
| 26-Nov-24 | 27.8 | 12.8 | 45 | 43 | 0 | 0 |
| 27-Nov-24 | 29 | 14.5 | 65 | 39 | 0 | 0 |
| 28-Nov-24 | 27.4 | 14 | 60 | 100 | 0 | 0 |
| 29-Nov-24 | 28 | 13.2 | 70 | 58 | 0 | 0 |
| 30-Nov-24 | 29 | 15 | 73 | 60 | 0 | 0 |
| 01-Dec-24 | 27.4 | 16.8 | 71 | 65 | 0 | 0 |
| 02-Dec-24 | 29.2 | 19 | 85 | 63 | 0 | 0 |
| 03-Dec-24 | 27.4 | 20.6 | 95 | 77 | 1.8 | 0 |
| 04-Dec-24 | 26.6 | 20.5 | 91 | 80 | 1.2 | 0 |
| 05-Dec-24 | 29.2 | 18 | 89 | 61 | 0 | 0 |
| 06-Dec-24 | 29.6 | 19.8 | 75 | 72 | 0 | 0 |
| 07-Dec-24 | 30.4 | 19 | 85 | 67 | 0 | 0 |
| 08-Dec-24 | 30 | 20.6 | 87 | 57 | 1 | 0 |
| 09-Dec-24 | 29 | 18.6 | 88 | 84 | 0 | 0 |
| 10-Dec-24 | 29.6 | 20 | 88 | 63 | 0.6 | 0 |
| 11-Dec-24 | 29.4 | 16.4 | 86 | 63 | 0 | 0 |
| 12-Dec-24 | 28.2 | 14.4 | 74 | 56 | 0 | 0 |
| 13-Dec-24 | 28.6 | 15.5 | 84 | 50 | 0 | 0 |
| 14-Dec-24 | 27.6 | 15.8 | 80 | 67 | 0 | 0 |
| 15-Dec-24 | 27.8 | 13 | 87 | 53 | 0 | 0 |
| 16-Dec-24 | 27 | 11.8 | 72 | 49 | 0 | 0 |
| 17-Dec-24 | 27 | 10.2 | 65 | 49 | 0 | 0 |
| 18-Dec-24 | 28 | 10 | 56 | 40 | 0 | 0 |
| 19-Dec-24 | 29 | 13.2 | 71 | 45 | 0 | 0 |
| 20-Dec-24 | 29.2 | 16.4 | 76 | 61 | 0 | 0 |
| 21-Dec-24 | 29.2 | 16.8 | 81 | 47 | 0 | 0 |
| 22-Dec-24 | 30 | 13.8 | 89 | 30 | 0 | 0 |
| 23-Dec-24 | 28.6 | 15.6 | 84 | 62 | 0 | 0 |
| 24-Dec-24 | 28.2 | 16.5 | 80 | 56 | 0 | 0 |
| 25-Dec-24 | 28.8 | 17.2 | 84 | 55 | 0 | 0 |
| 26-Dec-24 | 28.2 | 17 | 88 | 55 | 0 | 0 |
| 27-Dec-24 | 26.4 | 19.5 | 90 | 74 | 0 | 0 |
| 28-Dec-24 | 26 | 18.5 | 86 | 84 | 0 | 0 |
| 29-Dec-24 | 29.6 | 16.5 | 86 | 58 | 0 | 0 |
| 30-Dec-24 | 29.2 | 14.5 | 78 | 58 | 0 | 0 |
| 31-Dec-24 | 29.6 | 12.8 | 73 | 62 | 0 | 0 |
| 01-Jan-25 | 29.8 | 12.5 | 81 | 51 | 0 | 0 |
| 02-Jan-25 | 28.8 | 13.6 | 83 | 56 | 0 | 0 |
| 03-Jan-25 | 28.8 | 12.6 | 85 | 60 | 0 | 0 |
| 04-Jan-25 | 28.8 | 10.6 | 86 | 41 | 0 | 0 |
| 05-Jan-25 | 29 | 13 | 77 | 44 | 0 | 0 |
| 06-Jan-25 | 29 | 11.4 | 80 | 38 | 0 | 0 |
| 07-Jan-25 | 29 | 11.4 | 69 | 39 | 0 | 0 |
| 08-Jan-25 | 28.2 | 12.5 | 75 | 48 | 0 | 0 |
| 09-Jan-25 | 28.2 | 11 | 76 | 51 | 0 | 0 |
| 10-Jan-25 | 27.6 | 11.8 | 82 | 54 | 0 | 0 |
| 11-Jan-25 | 27.6 | 14.4 | 90 | 64 | 0 | 0 |
| 12-Jan-25 | 29.2 | 16.6 | 87 | 55 | 0 | 0 |
| 13-Jan-25 | 30.2 | 16.2 | 86 | 47 | 0 | 0 |
| 14-Jan-25 | 30 | 16 | 88 | 47 | 0 | 0 |
| 15-Jan-25 | 30.2 | 16.6 | 85 | 52 | 0 | 0 |
| 16-Jan-25 | 29.6 | 17 | 80 | 54 | 0 | 0 |
| 17-Jan-25 | 29.8 | 15 | 90 | 56 | 0 | 0 |
| 18-Jan-25 | 28.8 | 14.6 | 84 | 59 | 0 | 0 |
| 19-Jan-25 | 26.2 | 13.5 | 83 | 49 | 0 | 0 |
| 20-Jan-25 | 30 | 14.2 | 84 | 48 | 0 | 0 |
| 21-Jan-25 | 30 | 13.5 | 74 | 51 | 0 | 0 |
| 22-Jan-25 | 31 | 10.6 | 64 | 49 | 0 | 0 |
| 23-Jan-25 | 31.8 | 13.2 | 67 | 52 | 0 | 0 |
| 24-Jan-25 | 32 | 14.2 | 73 | 56 | 0 | 0 |
| 25-Jan-25 | 32.8 | 11.6 | 74 | 51 | 0 | 0 |
| 26-Jan-25 | 32.8 | 14 | 83 | 48 | 0 | 0 |
| 27-Jan-25 | 32 | 12.8 | 73 | 41 | 0 | 0 |
| 28-Jan-25 | 31.2 | 13 | 70 | 55 | 0 | 0 |
| 29-Jan-25 | 31.8 | 13 | 67 | 34 | 0 | 0 |
| 30-Jan-25 | 31.4 | 12.2 | 75 | 40 | 0 | 0 |
| 31-Jan-25 | 32.6 | 12.5 | 73 | 31 | 0 | 0 |
| 01-Feb-25 | 33.2 | 15.2 | 77 | 35 | 0 | 0 |
| 02-Feb-25 | 33.4 | 16 | 66 | 30 | 0 | 0 |
| 03-Feb-25 | 32.5 | 12.8 | 70 | 42 | 0 | 0 |
| 04-Feb-25 | 33 | 12.5 | 61 | 37 | 0 | 0 |
| 05-Feb-25 | 34 | 14.5 | 62 | 44 | 0 | 0 |
| 06-Feb-25 | 32.8 | 15 | 67 | 49 | 0 | 0 |
| 07-Feb-25 | 33.2 | 15.8 | 66 | 29 | 0 | 0 |
| 08-Feb-25 | 33.2 | 17.5 | 69 | 28 | 0 | 0 |
| 09-Feb-25 | 33 | 17.2 | 67 | 31 | 0 | 0 |
| 10-Feb-25 | 32.6 | 16.8 | 75 | 29 | 0 | 0 |
| 11-Feb-25 | 32.8 | 15.8 | 69 | 30 | 0 | 0 |
| 12-Feb-25 | 33 | 15.4 | 64 | 27 | 0 | 0 |
| 13-Feb-25 | 33.6 | 16 | 61 | 19 | 0 | 0 |
| 14-Feb-25 | 34.6 | 16.6 | 59 | 24 | 0 | 0 |
| 15-Feb-25 | 34.6 | 15.5 | 60 | 18 | 0 | 0 |
| 16-Feb-25 | 34 | 14.8 | 55 | 19 | 0 | 0 |
| 17-Feb-25 | 34.6 | 14.8 | 59 | 21 | 0 | 0 |
| 18-Feb-25 | 34.6 | 16.5 | 67 | 26 | 0 | 0 |
| 19-Feb-25 | 34.6 | 18.2 | 82 | 32 | 0 | 0 |
| 20-Feb-25 | 34 | 19 | 74 | 34 | 0 | 0 |
| 21-Feb-25 | 34.5 | 18.5 | 70 | 37 | 0 | 0 |
| 22-Feb-25 | 35 | 19.2 | 73 | 40 | 0 | 0 |
| 23-Feb-25 | 34.4 | 19 | 76 | 39 | 0 | 0 |
| 24-Feb-25 | 34.2 | 16.8 | 72 | 38 | 0 | 0 |
| 25-Feb-25 | 33.6 | 17 | 76 | 24 | 0 | 0 |
| 26-Feb-25 | 33.2 | 15 | 65 | 34 | 0 | 0 |
| 27-Feb-25 | 33.6 | 15.5 | 61 | 36 | 0 | 0 |
| 28-Feb-25 | 33.6 | 16 | 66 | 25 | 0 | 0 |
| 01-Mar-25 | 33.8 | 18.4 | 71 | 23 | 0 | 0 |
| 02-Mar-25 | 34.6 | 15.8 | 67 | 27 | 0 | 0 |
| 03-Mar-25 | 35.2 | 17.6 | 69 | 24 | 0 | 0 |
| 04-Mar-25 | 35.6 | 18.8 | 81 | 34 | 0 | 0 |
| 05-Mar-25 | 36 | 18.4 | 77 | 35 | 0 | 0 |
| 06-Mar-25 | 35.2 | 18.6 | 74 | 34 | 0 | 0 |
| 07-Mar-25 | 35.2 | 16 | 66 | 15 | 0 | 0 |
| 08-Mar-25 | 36.2 | 15.5 | 63 | 24 | 0 | 0 |
| 09-Mar-25 | 36.6 | 18.2 | 74 | 23 | 0 | 0 |
| 10-Mar-25 | 36.4 | 17.6 | 70 | 37 | 0 | 0 |
| 11-Mar-25 | 35.2 | 16.8 | 71 | 36 | 0 | 0 |
| 12-Mar-25 | 35.6 | 17 | 73 | 33 | 0 | 0 |
| 13-Mar-25 | 36.2 | 20 | 79 | 37 | 0 | 0 |
| 14-Mar-25 | 36.6 | 20.8 | 83 | 35 | 0 | 0 |
| 15-Mar-25 | 36.6 | 21.4 | 85 | 30 | 0 | 0 |
| 16-Mar-25 | 38.2 | 19.4 | 75 | 29 | 0 | 0 |
