# Supplementary material for: Genetic mapping and candidate gene identification for key physiological traits associated with heat tolerance in wheat (Triticum aestivum L.) using a MAGIC population
Source: PLoS One. 2026 Jan 2;21(1):e0339966. doi: 10.1371/journal.pone.0339966 (PMC12758712; doi:10.1371/journal.pone.0339966)
Supplement: S9 Table — (DOCX) [file pone.0339966.s009.docx]

**S9 Table.** **Meteorological data for the 2024-25 Rabi season at the Pune station.**

| Date | Max.Temp (°C) | Min.Temp (°C) | RH Max (%) | RH Min (%) | Rainfall (mm) | NRD (days) |
| --- | --- | --- | --- | --- | --- | --- |
| 1. Nov. 2024 | 33 | 22.2 | 94.41 | 91.38 | 0 | 0 |
| 2. Nov. 2024 | 32.5 | 20 | 91.63 | 90.85 | 0 | 0 |
| 3. Nov. 2024 | 31 | 20 | 87.8 | 86.12 | 0 | 0 |
| 4. Nov. 2024 | 31.8 | 17.2 | 98.19 | 91.47 | 0 | 0 |
| 5. Nov. 2024 | 32 | 18 | 90.34 | 88.89 | 0 | 0 |
| 6. Nov. 2024 | 32.2 | 17 | 94.59 | 91.31 | 0 | 0 |
| 7. Nov. 2024 | 31.5 | 15.5 | 89.08 | 87.17 | 0 | 0 |
| 8. Nov. 2024 | 31.2 | 15.8 | 95.37 | 93.14 | 0 | 0 |
| 9. Nov. 2024 | 31.6 | 15.6 | 92.59 | 91.92 | 0 | 0 |
| 10. Nov. 2024 | 31.8 | 15.8 | 95.48 | 90.44 | 0 | 0 |
| 11. Nov. 2024 | 30.2 | 15.9 | 94.45 | 91.6 | 0 | 0 |
| 12. Nov. 2024 | 30.5 | 14.5 | 90.58 | 90.53 | 0 | 0 |
| 13. Nov. 2024 | 31.2 | 15 | 84.47 | 86.96 | 0 | 0 |
| 14. Nov. 2024 | 31 | 16.3 | 95.37 | 94.4 | 0 | 0 |
| 15. Nov. 2024 | 30.2 | 22 | 84.34 | 86.06 | 0 | 0 |
| 16. Nov. 2024 | 31.1 | 21.7 | 90.95 | 91.17 | 0 | 0 |
| 17. Nov. 2024 | 31 | 20.3 | 84.43 | 86.11 | 0 | 0 |
| 18. Nov. 2024 | 30.1 | 14.1 | 94.27 | 92.89 | 0 | 0 |
| 19. Nov. 2024 | 30.2 | 12.5 | 89.25 | 88.83 | 0 | 0 |
| 20. Nov. 2024 | 29 | 11.5 | 92.18 | 91.43 | 0 | 0 |
| 21. Nov. 2024 | 29.7 | 11.4 | 96.84 | 93.9 | 0 | 0 |
| 22. Nov. 2024 | 28.8 | 12.2 | 91.03 | 98.2 | 0 | 0 |
| 23. Nov. 2024 | 30.2 | 12.5 | 94.98 | 92.29 | 0 | 0 |
| 24. Nov. 2024 | 29.8 | 12.5 | 92.49 | 95.09 | 0 | 0 |
| 25. Nov. 2024 | 28.5 | 11.5 | 97.9 | 94.59 | 0 | 0 |
| 26. Nov. 2024 | 27.5 | 10.8 | 97.81 | 90.99 | 0 | 0 |
| 27. Nov. 2024 | 28.6 | 9.8 | 91.73 | 90.04 | 0 | 0 |
| 28. Nov. 2024 | 28.1 | 9.3 | 97.75 | 92.95 | 0 | 0 |
| 29. Nov. 2024 | 27.5 | 8.5 | 96.59 | 93.13 | 0 | 0 |
| 30. Nov. 2024 | 27.8 | 10 | 95.52 | 91.18 | 0 | 0 |
| 1. Dec. 2024 | 28 | 14.2 | 83.63 | 87.06 | 0 | 0 |
| 2. Dec. 2024 | 29.5 | 21.1 | 90.12 | 89.87 | 0 | 0 |
| 3. Dec. 2024 | 30.1 | 22.7 | 89.8 | 91.48 | 0 | 0 |
| 4. Dec. 2024 | 30.8 | 22.7 | 95.84 | 94.64 | 0 | 0 |
| 5. Dec. 2024 | 32.5 | 18.5 | 95.59 | 94.18 | 0 | 0 |
| 6. Dec. 2024 | 31.6 | 20.2 | 97.4 | 95.55 | 0 | 0 |
| 7. Dec. 2024 | 31.8 | 19 | 98.23 | 94.88 | 0 | 0 |
| 8. Dec. 2024 | 33.2 | 17 | 95.5 | 94.9 | 0 | 0 |
| 9. Dec. 2024 | 33.2 | 14 | 87.18 | 89.35 | 0 | 0 |
| 10. Dec. 2024 | 33 | 12.8 | 90.05 | 89.24 | 0 | 0 |
| 11. Dec. 2024 | 33.2 | 13.8 | 92.33 | 90.92 | 0 | 0 |
| 12. Dec. 2024 | 33 | 12.7 | 95.01 | 92.36 | 0 | 0 |
| 13. Dec. 2024 | 33 | 12 | 97.92 | 97.1 | 0 | 0 |
| 14. Dec. 2024 | 33.5 | 9.5 | 73.09 | 78.8 | 0 | 0 |
| 15. Dec. 2024 | 33 | 8.2 | 94.46 | 91.13 | 0 | 0 |
| 16. Dec. 2024 | 28.8 | 7.5 | 93.84 | 90.88 | 0 | 0 |
| 17. Dec. 2024 | 29.1 | 7.8 | 97.57 | 94.73 | 0 | 0 |
| 18. Dec. 2024 | 29.3 | 9 | 98.81 | 95.08 | 0 | 0 |
| 19. Dec. 2024 | 29.5 | 9.1 | 102.37 | 100.28 | 0 | 0 |
| 20. Dec. 2024 | 29.8 | 9.5 | 96.64 | 89.48 | 0 | 0 |
| 21. Dec. 2024 | 30.5 | 15 | 103.96 | 84.92 | 0 | 0 |
| 22. Dec. 2024 | 29.3 | 14.9 | 103 | 80.25 | 0 | 0 |
| 23. Dec. 2024 | 28.3 | 15.4 | 101.99 | 94.79 | 0 | 0 |
| 24. Dec. 2024 | 29 | 14.7 | 98.07 | 95 | 0 | 0 |
| 25. Dec. 2024 | 30 | 17.8 | 93.57 | 91.69 | 0 | 0 |
| 26. Dec. 2024 | 30.2 | 19 | 100 | 95.71 | 0 | 0 |
| 27. Dec. 2024 | 30.2 | 18.7 | 96.51 | 95.42 | 0 | 0 |
| 28. Dec. 2024 | 31.4 | 17.4 | 99.08 | 97.55 | 0 | 0 |
| 29. Dec. 2024 | 31.4 | 18 | 98.17 | 93.35 | 0 | 0 |
| 30. Dec. 2024 | 30.1 | 16 | 97.17 | 94.89 | 0 | 0 |
| 31. Dec. 2024 | 31.4 | 14.7 | 92.12 | 95.02 | 0 | 0 |
| 1. Jan. 2025 | 31.4 | 14.5 | 98 | 92.81 | 0 | 0 |
| 2. Jan. 2025 | 31.5 | 12.7 | 95.08 | 95.05 | 0 | 0 |
| 3. Jan. 2025 | 31 | 10.5 | 86.24 | 86.16 | 0 | 0 |
| 4. Jan. 2025 | 30.2 | 8.8 | 87.07 | 87.88 | 0 | 0 |
| 5. Jan. 2025 | 31 | 10 | 87.64 | 86.32 | 0 | 0 |
| 6. Jan. 2025 | 32 | 10.2 | 97.93 | 96.32 | 0 | 0 |
| 7. Jan. 2025 | 31 | 13 | 92.14 | 87.72 | 0 | 0 |
| 8. Jan. 2025 | 31 | 12 | 92.94 | 89.03 | 0 | 0 |
| 9. Jan. 2025 | 30.7 | 10.5 | 88.17 | 88.11 | 0 | 0 |
| 10. Jan. 2025 | 31 | 10.5 | 98.98 | 93.98 | 0 | 0 |
| 11. Jan. 2025 | 30.8 | 11 | 98.98 | 95.52 | 0 | 0 |
| 12. Jan. 2025 | 31 | 15.7 | 95.3 | 92.34 | 0 | 0 |
| 13. Jan. 2025 | 29.2 | 16.2 | 98.09 | 95.4 | 0 | 0 |
| 14. Jan. 2025 | 30.2 | 15.4 | 96.17 | 90.12 | 0 | 0 |
| 15. Jan. 2025 | 29.4 | 15.4 | 93.37 | 99.06 | 0 | 0 |
| 16. Jan. 2025 | 30.8 | 14.3 | 98.05 | 95.57 | 0 | 0 |
| 17. Jan. 2025 | 29.2 | 15.3 | 93.37 | 93.01 | 0 | 0 |
| 18. Jan. 2025 | 28.7 | 12.2 | 92.89 | 91.68 | 0 | 0 |
| 19. Jan. 2025 | 29.5 | 12.7 | 93.07 | 91.18 | 0 | 0 |
| 20. Jan. 2025 | 30 | 11.8 | 92.79 | 93.56 | 0 | 0 |
| 21. Jan. 2025 | 31.2 | 12.5 | 92.01 | 92.34 | 0 | 0 |
| 22. Jan. 2025 | 32.2 | 12.2 | 89.31 | 88.16 | 0 | 0 |
| 23. Jan. 2025 | 32.8 | 12.4 | 92.98 | 91.01 | 0 | 0 |
| 24. Jan. 2025 | 35.5 | 13 | 94.08 | 90.01 | 0 | 0 |
| 25. Jan. 2025 | 33.8 | 13.5 | 91.44 | 90.7 | 0 | 0 |
| 26. Jan. 2025 | 32.5 | 13.1 | 95.09 | 93.61 | 0 | 0 |
| 27. Jan. 2025 | 32.8 | 13.5 | 93.28 | 92.89 | 0 | 0 |
| 28. Jan. 2025 | 33.8 | 13 | 90.58 | 87.5 | 0 | 0 |
| 29. Jan. 2025 | 33.8 | 14.3 | 92.51 | 90.51 | 0 | 0 |
| 30. Jan. 2025 | 34.3 | 14 | 90.58 | 89.51 | 0 | 0 |
| 31. Jan. 2025 | 33.2 | 15 | 97.17 | 96.51 | 0 | 0 |
| 1. Feb. 2025 | 34.3 | 16.7 | 98.98 | 93.18 | 0 | 0 |
| 2. Feb. 2025 | 34 | 14 | 87.37 | 87 | 0 | 0 |
| 3. Feb. 2025 | 34 | 15.2 | 90.87 | 89.57 | 0 | 0 |
| 4. Feb. 2025 | 34.2 | 13.4 | 97.62 | 93.43 | 0 | 0 |
| 5. Feb. 2025 | 34.2 | 13.4 | 82.8 | 83.97 | 0 | 0 |
| 6. Feb. 2025 | 34.5 | 14.9 | 85.66 | 88.04 | 0 | 0 |
| 7. Feb. 2025 | 34.6 | 14 | 95.48 | 99.32 | 0 | 0 |
| 8. Feb. 2025 | 34.8 | 16.2 | 91.89 | 90.95 | 0 | 0 |
| 9. Feb. 2025 | 34.8 | 15.7 | 92.63 | 89.43 | 0 | 0 |
| 10. Feb. 2025 | 34.8 | 17.1 | 91.27 | 90.11 | 0 | 0 |
| 11. Feb. 2025 | 33.6 | 17 | 91.05 | 81.55 | 0 | 0 |
| 12. Feb. 2025 | 34.5 | 15 | 86.36 | 86.59 | 0 | 0 |
| 13. Feb. 2025 | 33 | 12.5 | 81.49 | 84.4 | 0 | 0 |
| 14. Feb. 2025 | 32.5 | 12.4 | 81.25 | 83.63 | 0 | 0 |
| 15. Feb. 2025 | 34.2 | 12 | 81.97 | 83.44 | 0 | 0 |
| 16. Feb. 2025 | 34.1 | 12 | 97.92 | 92.21 | 0 | 0 |
| 17. Feb. 2025 | 34 | 12.5 | 87.75 | 86.99 | 0 | 0 |
| 18. Feb. 2025 | 35 | 12.4 | 88.28 | 88.52 | 0 | 0 |
| 19. Feb. 2025 | 34.7 | 14.8 | 85.66 | 84.05 | 0 | 0 |
| 20. Feb. 2025 | 36 | 15.2 | 93.49 | 87.66 | 0 | 0 |
| 21. Feb. 2025 | 35 | 15 | 72.21 | 75.69 | 0 | 0 |
| 22. Feb. 2025 | 35.2 | 14 | 64.87 | 72.59 | 0 | 0 |
| 23. Feb. 2025 | 35 | 13.4 | 81.63 | 80.94 | 0 | 0 |
| 24. Feb. 2025 | 34.5 | 14 | 83.63 | 85.45 | 0 | 0 |
| 25. Feb. 2025 | 33.2 | 13.5 | 84.67 | 84.28 | 0 | 0 |
| 26. Feb. 2025 | 33.5 | 13.8 | 82.2 | 85.78 | 0 | 0 |
| 27. Feb. 2025 | 33.6 | 14.1 | 78.5 | 81.55 | 0 | 0 |
| 28. Feb. 2025 | 34.6 | 15 | 88.46 | 87.22 | 0 | 0 |
| 1. Mar. 2025 | 35 | 15.2 | 84.44 | 87.47 | 0 | 0 |
| 2. Mar. 2025 | 36.3 | 16.5 | 78.45 | 81.71 | 0 | 0 |
| 3. Mar. 2025 | 36 | 16.2 | 81.21 | 84.12 | 0 | 0 |
| 4. Mar. 2025 | 36.4 | 15.2 | 78.92 | 82.22 | 0 | 0 |
| 5. Mar. 2025 | 36.5 | 14.6 | 76.8 | 80.67 | 0 | 0 |
| 6. Mar. 2025 | 36.2 | 13.1 | 76.23 | 82.6 | 0 | 0 |
| 7. Mar. 2025 | 36 | 11.5 | 86.03 | 89.38 | 0 | 0 |
| 8. Mar. 2025 | 37.2 | 12.2 | 87.59 | 90.69 | 0 | 0 |
| 9. Mar. 2025 | 37.8 | 13.4 | 88.74 | 88.68 | 0 | 0 |
| 10. Mar. 2025 | 35.5 | 15.1 | 78.53 | 51.18 | 0 | 0 |
| 11. Mar. 2025 | 35.2 | 16.4 | 63 | 41.31 | 0 | 0 |
| 12. Mar. 2025 | 36.8 | 16.5 | 58.3 | 36.92 | 0 | 0 |
| 13. Mar. 2025 | 37.8 | 17.5 | 72.4 | 42.61 | 0 | 0 |
| 14. Mar. 2025 | 37.9 | 18 | 84.8 | 57.1 | 0 | 0 |
| 15. Mar. 2025 | 37.8 | 18.5 | 100 | 100 | 0 | 0 |
| 16. Mar. 2025 | 37.5 | 16.7 | 67.76 | 45.17 | 0 | 0 |
| 17. Mar. 2025 | 37.8 | 17.2 | 67.54 | 64.34 | 0 | 0 |
| 18. Mar. 2025 | 37.5 | 16 | 70.81 | 73.59 | 0 | 0 |
| 19. Mar. 2025 | 37.8 | 16.2 | 77.11 | 72.34 | 0 | 0 |
| 20. Mar. 2025 | 36.2 | 16.5 | 68.11 | 69.88 | 0 | 0 |
| 21. Mar. 2025 | 35.5 | 16.8 | 74.4 | 77.99 | 0 | 0 |
| 22. Mar. 2025 | 37 | 15.5 | 100 | 65.97 | 0 | 0 |
| 23. Mar. 2025 | 37.5 | 17 | 75.6 | 79.26 | 0 | 0 |
| 24. Mar. 2025 | 36.8 | 17 | 70.1 | 75.34 | 0 | 0 |
| 25. Mar. 2025 | 37.7 | 16.8 | 85.48 | 87.31 | 0 | 0 |
| 26. Mar. 2025 | 38.3 | 21 | 84.67 | 86.68 | 0 | 0 |
| 27. Mar. 2025 | 37 | 20.5 | 81.89 | 83.05 | 0 | 0 |
| 28. Mar. 2025 | 38.8 | 19.2 | 76.45 | 77.73 | 0 | 0 |
| 29. Mar. 2025 | 38.7 | 20.23 | 100 | 100 | 0 | 0 |
| 30. Mar. 2025 | 39.12 | 24 | 82.44 | 86.51 | 0 | 0 |
| 31. Mar. 2025 | 38.5 | 21.2 | 100 | 100 | 0 | 0 |
| 1. Apr. 2025 | 39 | 22.8 | 92.34 | 84.36 | 0 | 0 |
| 2. Apr. 2025 | 38.5 | 21.2 | 87.75 | 72.3 | 4.6 | 1 |
| 3. Apr. 2025 | 39.1 | 20.2 | 79.36 | 85.04 | 0 | 0 |
| 4. Apr. 2025 | 35.2 | 17 | 81.42 | 85.71 | 0 | 0 |
| 5. Apr. 2025 | 37.5 | 17.2 | 73.37 | 73.93 | 0 | 0 |
| 6. Apr. 2025 | 38.6 | 18.23 | 100 | 100 | 0 | 0 |
| 7. Apr. 2025 | 39.3 | 16.5 | 69.75 | 76.66 | 0 | 0 |
| 8. Apr. 2025 | 40 | 19.2 | 81.33 | 85.21 | 0 | 0 |
| 9. Apr. 2025 | 40 | 23.2 | 88.22 | 89.81 | 0 | 0 |
| 10. Apr. 2025 | 42.2 | 24.5 | 85.55 | 87.77 | 0 | 0 |
| 11. Apr. 2025 | 38.5 | 20.9 | 88.39 | 88.77 | 0 | 0 |
| 12. Apr. 2025 | 38.8 | 22.5 | 87.59 | 89.22 | 0 | 0 |
| 13. Apr. 2025 | 39.12 | 21.7 | 83.27 | 87.72 | 1.8 | 0 |
| 14. Apr. 2025 | 39 | 24.2 | 91.31 | 92.79 | 0 | 0 |
| 15. Apr. 2025 | 39.8 | 25 | 51.92 | 62.75 | 0 | 0 |
| 16. Apr. 2025 | 40 | 24.7 | 66.49 | 74.28 | 0 | 0 |
| 17. Apr. 2025 | 40.7 | 26.1 | 81.4 | 81.92 | 0 | 0 |
| 18. Apr. 2025 | 39.3 | 25.2 | 100 | 100 | 0 | 0 |
| 19. Apr. 2025 | 40.5 | 27 | 83.61 | 87.41 | 0 | 0 |
| 20. Apr. 2025 | 40.12 | 27.32 | 100 | 100 | 0 | 0 |
| 21. Apr. 2025 | 39.3 | 25.2 | 80.57 | 80.34 | 0 | 0 |
| 22. Apr. 2025 | 40.2 | 28 | 74.49 | 59.48 | 0 | 0 |
| 23. Apr. 2025 | 41 | 28.5 | 69.01 | 50.11 | 0 | 0 |
| 24. Apr. 2025 | 41.2 | 29 | 69.29 | 75.3 | 0 | 0 |
| 25. Apr. 2025 | 40 | 28.8 | 82.6 | 61.69 | 0 | 0 |
| 26. Apr. 2025 | 41 | 28 | 63.72 | 71.06 | 0 | 0 |
| 27. Apr. 2025 | 40.5 | 26.7 | 94.93 | 94.43 | 0 | 0 |
| 28. Apr. 2025 | 40 | 27.2 | 72.96 | 49.89 | 0 | 0 |
| 29. Apr. 2025 | 40.5 | 27.8 | 67.13 | 75.02 | 0 | 0 |
| 30. Apr. 2025 | 41.1 | 28 | 68.71 | 76.66 | 0 | 0 |
