# Supplementary material for: Genetic mapping and candidate gene identification for key physiological traits associated with heat tolerance in wheat (Triticum aestivum L.) using a MAGIC population
Source: PLoS One. 2026 Jan 2;21(1):e0339966. doi: 10.1371/journal.pone.0339966 (PMC12758712; doi:10.1371/journal.pone.0339966)
Supplement: S2 Fig — (DOCX) [file pone.0339966.s011.docx]

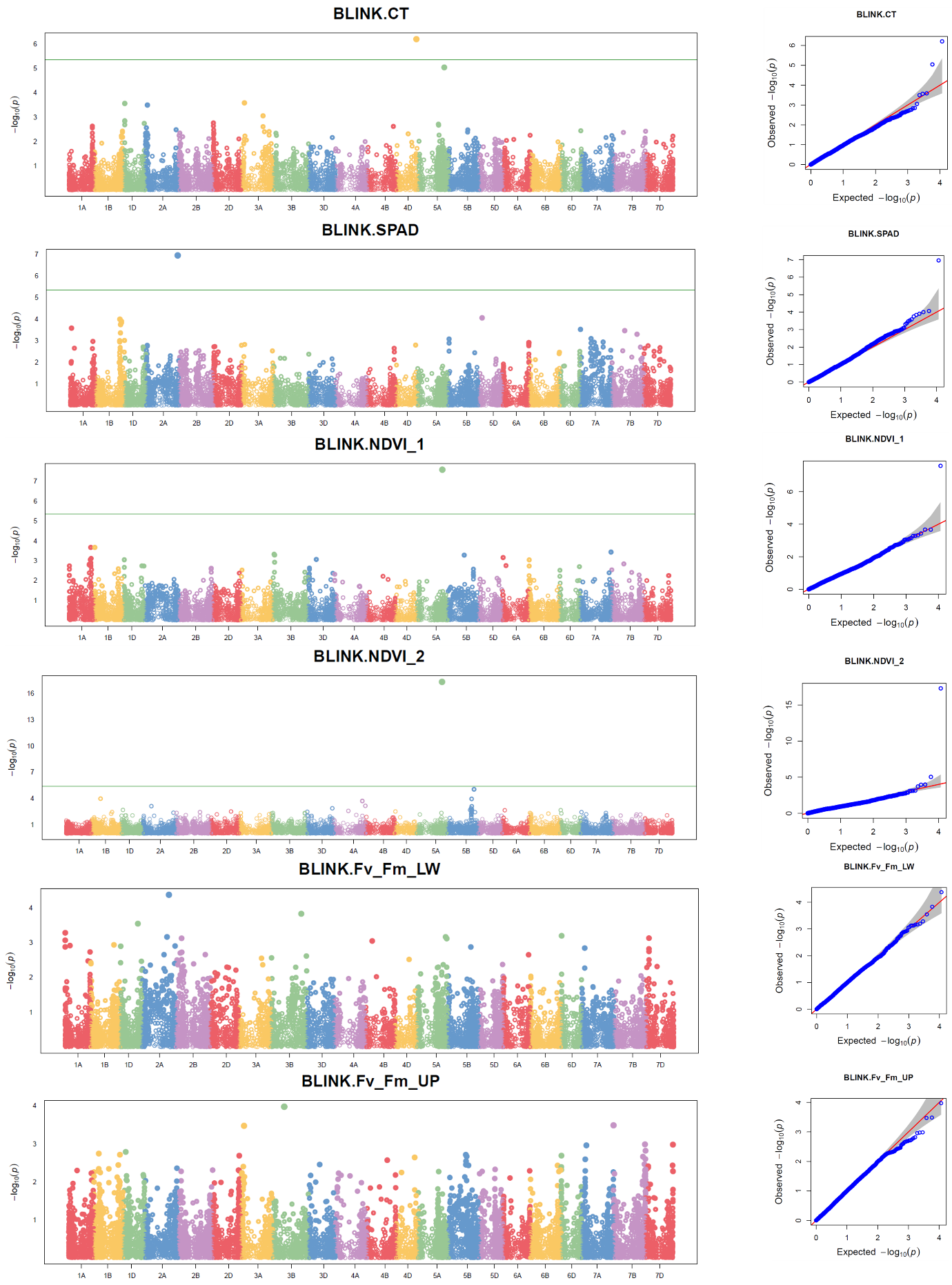


**S2 Figure.** Manhattan plots depicting SNP associations for physiological traits under LSIR condition across location during the 2024-25 season.
