# Supplementary material for: Genetic mapping and candidate gene identification for key physiological traits associated with heat tolerance in wheat (Triticum aestivum L.) using a MAGIC population
Source: PLoS One. 2026 Jan 2;21(1):e0339966. doi: 10.1371/journal.pone.0339966 (PMC12758712; doi:10.1371/journal.pone.0339966)
Supplement: S3 Fig — TraesCS1A02G098800(a), TraesCS1A02G099500(b), TraesCS1B02G093300(c), TraesCS5A02G077900(d), TraesCS5A02G078000(e) TraesCS7A02G143900 (f) and TraesCS7B02G083500 (g). (DOCX) [file pone.0339966.s012.docx]

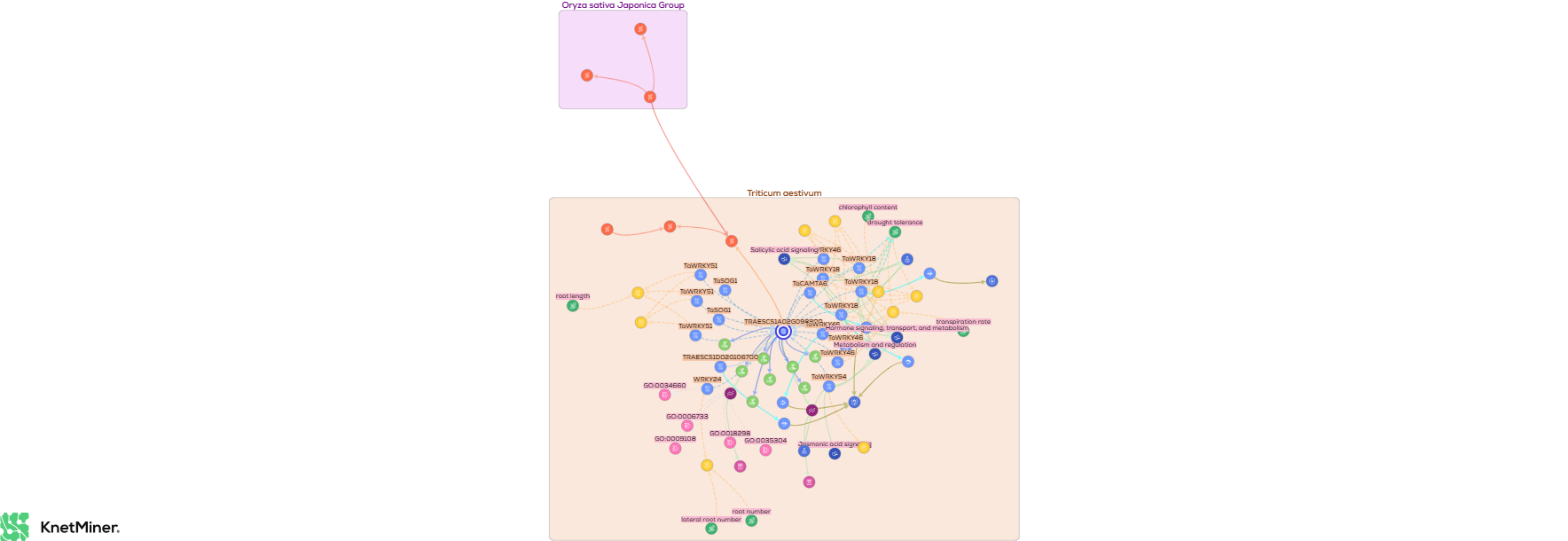


a

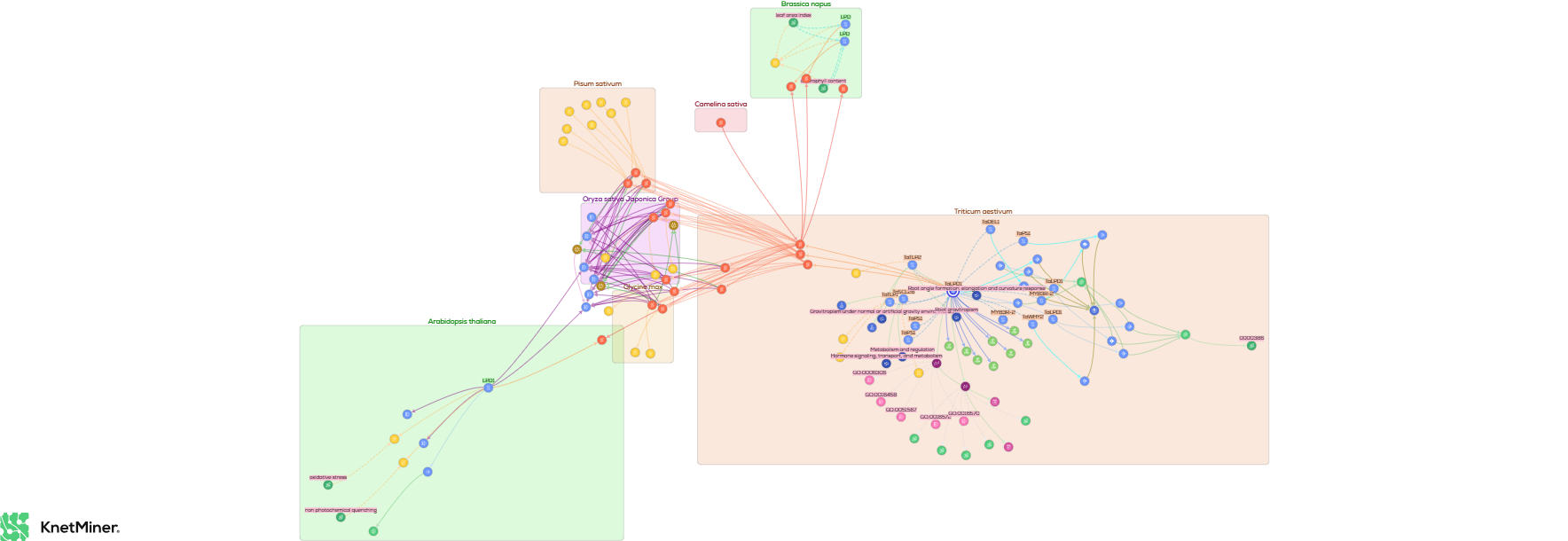


b

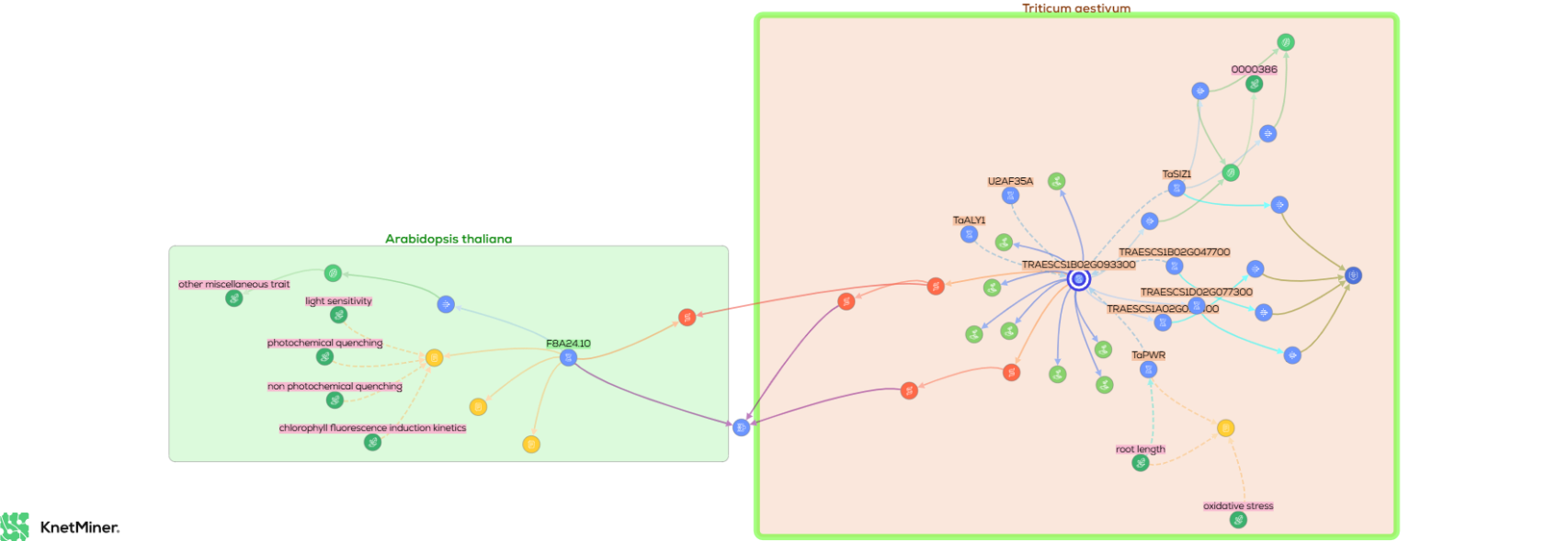


c

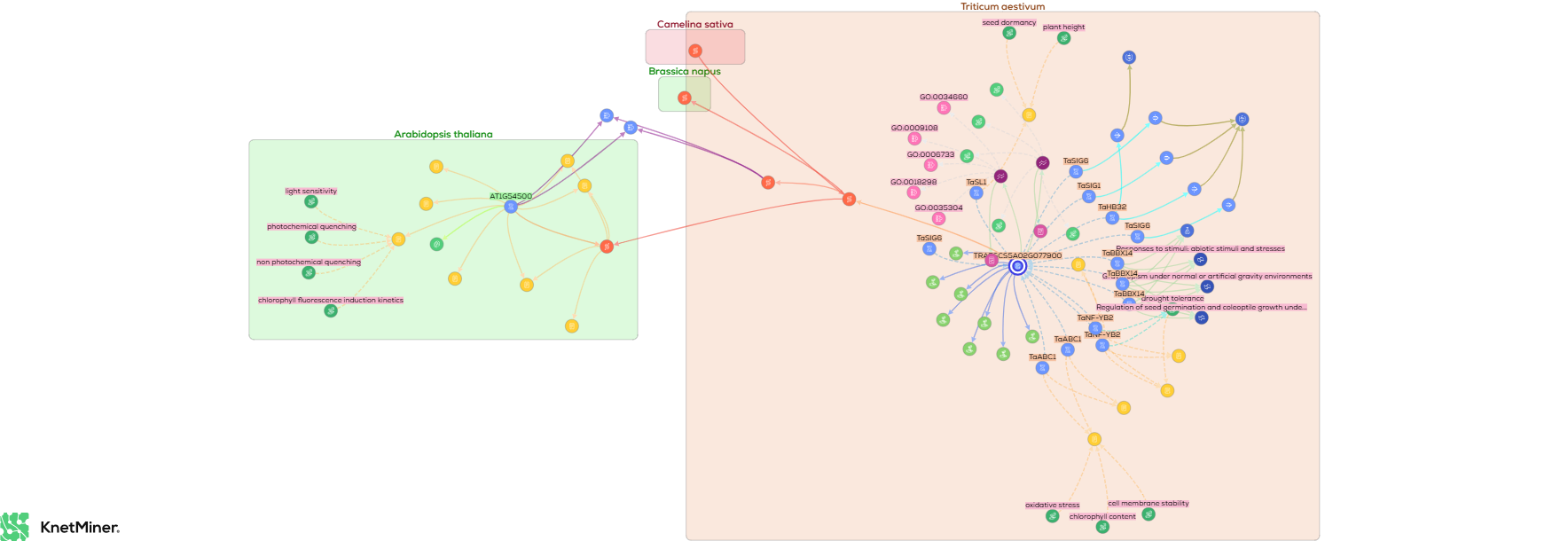


d

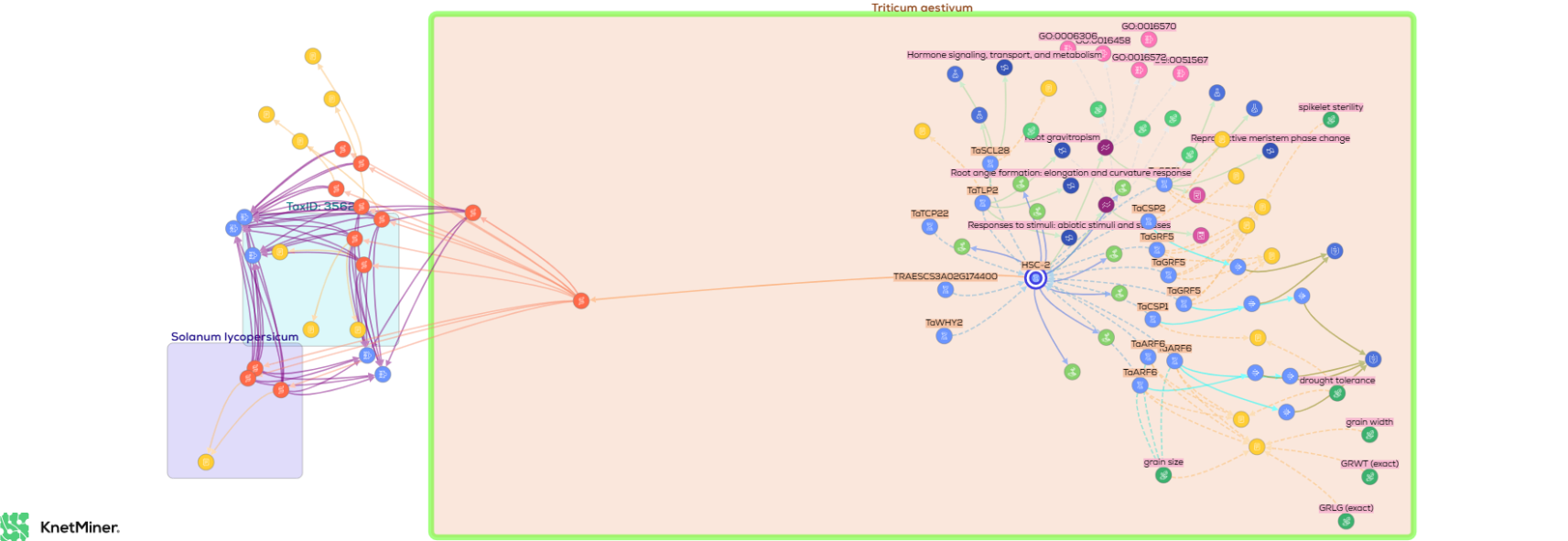


e

f

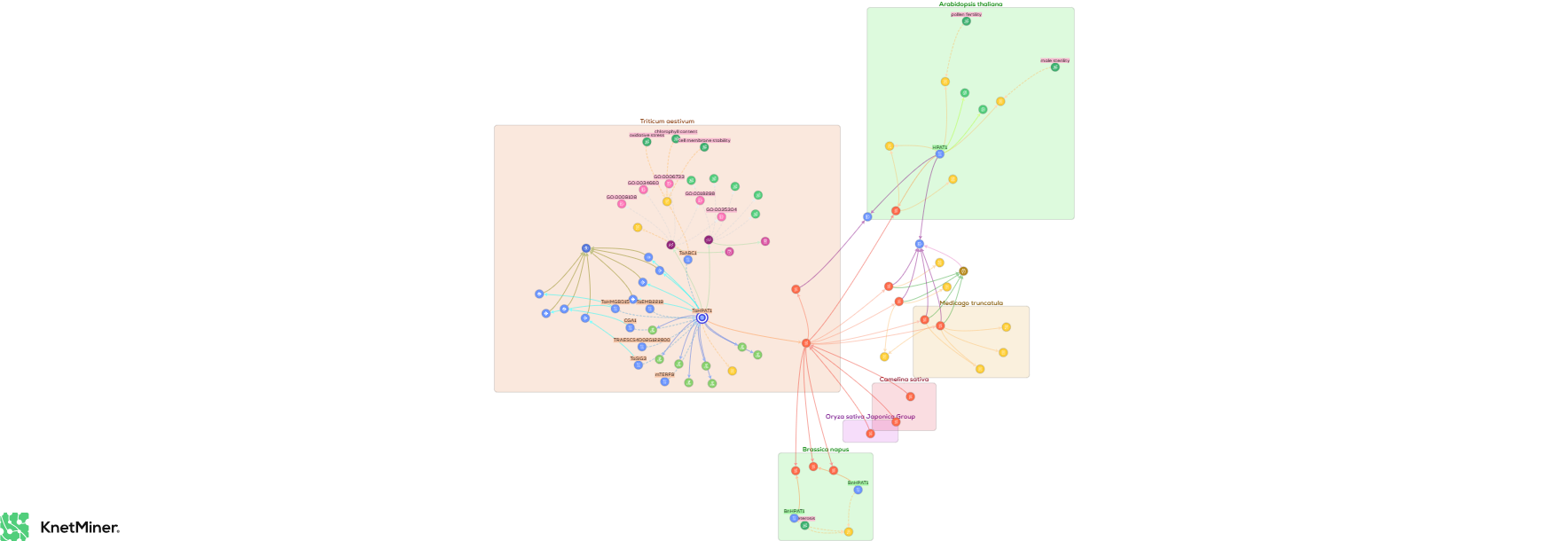


f

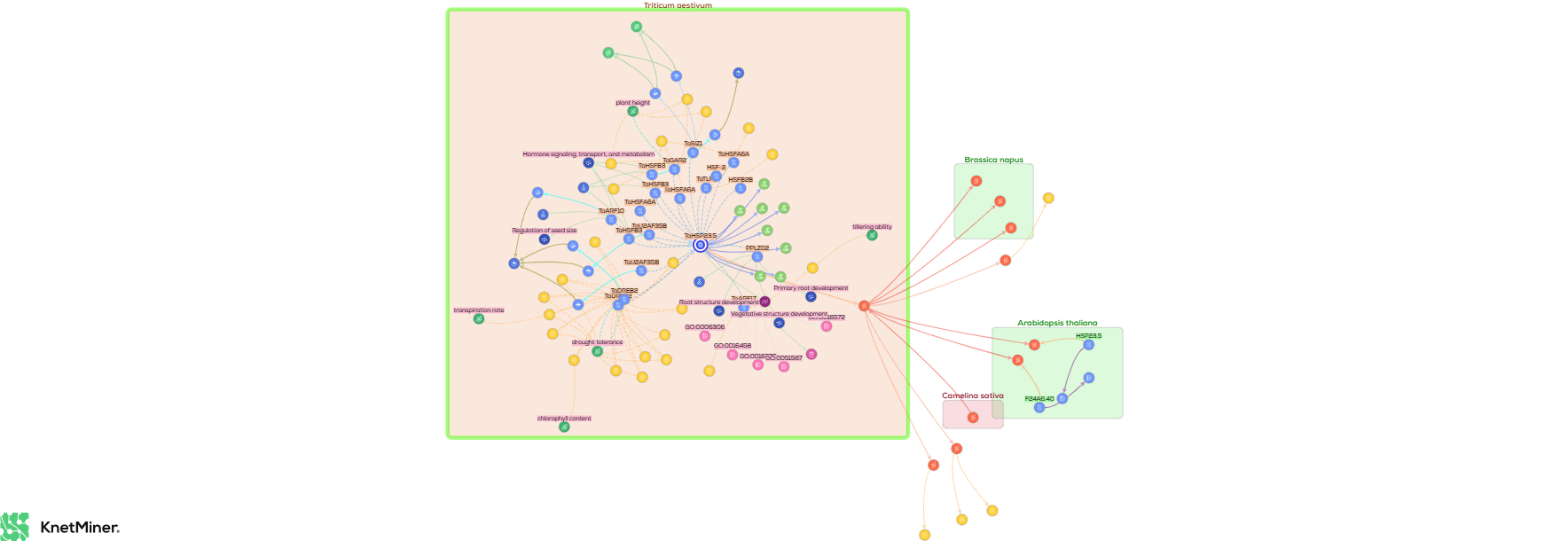


g

**S3 Figure** Gene regulatory networks of candidate genes associated with physiological traits in the MAGIC population. *TraesCS1A02G098800*(**a**), *TraesCS1A02G099500*(**b**), *TraesCS1B02G093300*(**c**), *TraesCS5A02G077900*(**d**), *TraesCS5A02G078000*(**e**) *TraesCS7A02G143900* (**f**) and *TraesCS7B02G083500* (**g**).
